# Tissue-Specific Expression of TIGIT, PD-1, TIM-3, and CD39 by γδ T Cells in Ovarian Cancer

**DOI:** 10.3390/cells11060964

**Published:** 2022-03-11

**Authors:** Pauline Weimer, Jasmin Wellbrock, Tabea Sturmheit, Leticia Oliveira-Ferrer, Yi Ding, Stephan Menzel, Marius Witt, Louisa Hell, Barbara Schmalfeldt, Carsten Bokemeyer, Walter Fiedler, Franziska Brauneck

**Affiliations:** 1 Department of Oncology, Hematology and Bone Marrow Transplantation with Section Pneumology, Hubertus Wald University Cancer Center, University Medical Center Hamburg-Eppendorf, 20251 Hamburg, Germany; pauline.weimer@stud.uke.uni-hamburg.de (P.W.); tms@2curex.com (T.S.); marius-p-j.witt@stud.uke.uni-hamburg.de (M.W.); c.bokemeyer@uke.de (C.B.); fiedler@uke.de (W.F.); 2 2cureX GmbH, 20251 Hamburg, Germany; lh@2curex.com; 3 Department of Gynecology, University Medical Center Hamburg-Eppendorf, 20251 Hamburg, Germany; ferrer@uke.de (L.O.-F.); yi.ding@stud.uke.uni-hamburg.de (Y.D.); b.schmalfeldt@uke.de (B.S.); 4 Mildred Scheel Cancer Career Center HaTriCS4, University Medical Center Hamburg-Eppendorf, 20251 Hamburg, Germany; s.menzel@uke.de; 5 Institute of Immunology, University Medical Center Hamburg-Eppendorf, 20251 Hamburg, Germany

**Keywords:** γδ T cells, ovarian cancer, TIL, ascites, TIGIT, PD-1, CD39, differentiation, co-expression

## Abstract

Phenotypic characterization of γδ T cells in the MALs (malignant ascites lymphocytes), TILs (tumor infiltrating lymphocytes), and PBLs (peripheral blood lymphocytes) of ovarian cancer (OvCA) patients is lacking. Therefore, we quantified γδ T cell prevalence in MAL, TIL, and PBL specimens from *n* = 18 OvCA patients and PBL from age-matched healthy donors (HD, *n* = 14). Multicolor flow cytometry was performed to evaluate the expression of inhibitory receptors (TIGIT, PD-1 and TIM-3), stimulatory receptors (Ox40), and purinergic ectoenzymes (CD39 and CD73) on γδ T cell subsets. We identified an abundant infiltration of Vδ1 T cells in the MALs and TILs. These cells varied in their differentiation: The majority of Vδ1 TILs displayed an effector memory (EM) phenotype, whereas Vδ1 MALs had a more mature phenotype of terminally differentiated effector memory cells (TEMRA) with high CD45RA expression. TIGIT and TIM-3 were abundantly expressed in both MALs and PBLs, whereas Vδ1 TILs exhibited the highest levels of PD-1, CD39, and Ox40. We also observed specific clusters on mature differentiation stages for the analyzed molecules. Regarding co-expression, Vδ1 TILs showed the highest levels of cells co-expressing TIGIT with PD-1 or CD39 compared to MALs and PBLs. In conclusion, the Vδ1 T cell population showed a high prevalence in the MALs and primary tumors of OvCA patients. Due to their (co-)expression of targetable immune receptors, in particular TIGIT with PD-1 and CD39 in TILs, Vδ1 T cell-based approaches combined with the inhibition of these targets might represent a promising strategy for OvCA.

## 1. Introduction

According to the SEER data base, ovarian cancer (OvCA) ranks fifth in cancer deaths among women with a rate of new cases of 10.9 per 100,000 and a death rate of 6.5 per 100,000. Due to the asymptomatic course of disease, most OvCA cases are diagnosed in an advanced stage [1,2]. In spite of multi-modal therapy (surgery and chemotherapy), the majority of patients succumb to their disease and the 5-year survival rate is below 45% [3].

γδ T cells interact via their T cell receptors (TCR), but also non-major histocompatibility complex (MHC)—restricted by the expression of natural killer cell receptors (NKRs) [4]. NKRs bind to surface proteins associated with disease or stress conditions on (malignant) cells [5]. These features make them independent of immune evasion mechanisms including downregulation of MHC presentation and of mutated epitopes [6,7,8]. Furthermore, their contribution to anti-tumor immune responses is explained by unique characteristics including migration to peripheral tissues rather than lymphoid organs, antigen-specificity, high clonal frequencies, and cytokine-dependent differentiation status that allows for rapid responses [9,10,11]. Although initial clinical trials are already testing γδ T cell-based chimeric antigen receptor T cells (CAR-T cells) or bispecific T cell engagers (BiTEs) [6], the expression of co-inhibitory targets on γδ T cells has been barely explored to this day. This knowledge may shed light onto their state of activation and exhaustion. Additionally, identification of checkpoint molecules may identify targets restoring γδ T cell-mediated cytotoxicity.

γδ T cells are subclassified based on their carried Vδ chain. Vδ2^+^ and Vγ9^+^ T cells constitute the majority (50–95%) of γδ T cells circulating in the blood [11]. In contrast, Vδ1-expressing T cells are most abundant in peripheral tissues, including infiltration of solid tumors [11,12].

Functionally, it has been demonstrated that both γδ populations can exert cytotoxic effector capacities by their TCR and natural killer group 2D (NKG2D) receptor signaling, resulting in secretion of interferon (IFN)-γ, tumor necrosis factor (TNF)-α, perforin, or granzymes [12,13,14].

On the other hand, immunosuppressive or tumor-promoting capabilities have also been described for γδ T cells, especially via secretion of IL-17 [15]. Furthermore, recruitment of immunosuppressive myeloid-derived suppressor cells (MDSCs) has been shown, mediated via secretion of IL-8, TNF, and granulocyte–macrophage colony-stimulating factor (GM-CSF) [16].

Assuming their biological relevance in human cancer, we phenotyped γδ T cells in OvCA, analyzing the lymphocytes of the peripheral blood (PBLs), malignant ascites (MALs), and tumor tissue (TILs) of patients with newly diagnosed OvCA. This study is focused on the expression of co-regulatory receptors such as the novel receptor T cell Ig and ITIM domain (TIGIT), programmed cell death protein-1 (PD-1), and the T cell immunoglobulin and mucin domain-containing protein 3 (TIM-3) on γδ T cells, which are important regulators of inflammatory responses by inhibiting CD3^+^ T cell effector activity [17,18,19]. Expression of these receptors has been shown to be negatively associated with cytokine production and/or proliferation of CD8^+^ and CD4^+^ T cells [20]. Furthermore, co-expression of multiple co-inhibitory receptors in TILs was identified as a characteristic of dysfunctional “exhausted” CD8^+^ TILs in different tumor entities [20,21]. Combined blockade of TIGIT or TIM-3 together with PD-1 additionally increased proliferation, cytokine production, and degranulation of CD8^+^ T cells [20]. In addition, we investigated Ox40 (also known as tumor necrosis factor receptors superfamily, member 40), which is mainly expressed by activated CD3^+^ T cells, thus regulating CD3^+^ T cell division, differentiation, and survival [22].

Another mechanism in the tumor environment leading to exhausted T cells is the increased production of adenosine via sequential hydrolysis of adenosine-triphosphate (ATP) by the ectonucleoside triphosphate diphosphohydrolase-1 (CD39) and the ecto-5’-nucleotidase (CD73) [23,24,25]. It has been shown that CD39 is especially expressed by CD8^+^ TILs and thereby defined exhaustion in these cells [23]. In contrast, CD73 is rather expressed by naïve CD8^+^ T cells and tumor cells [26,27]. Furthermore, increased anti-tumor immunity including cytotoxic T cell function was achieved by targeting the adenosine-generating enzymes CD39 and CD73 [28].

In conclusion, since all these molecules have an important role in the regulation of αβ T cells, it is very likely that they also have an important influence on the function of γδ T cells. By characterizing γδ T cells in OvCA in this study, we aimed to identify new suitable targets to increase the cytotoxic potential of these novel effector cells for ovarian cancer patients.

## 2. Materials and Methods

### 2.1. Patient Cohorts

PBLs, MALs, and TILs were collected from patients (*n* = 17, *n* = 18, and *n* = 9, respectively) with newly diagnosed high-grade serous ovarian cancer before the start of treatment. From nine patients, we obtained matched samples (PBLs and MALs), whereas from nine patients we collected triple matched samples (PBLs, MALs, and TILs). Due to low cell vitality, one PBL sample was excluded from the analysis. Moreover, we collected PBL specimens from healthy age-matched female donors (HDs, *n* = 14). The study was conducted in accordance with the provisions of the Declaration of Helsinki and was approved by the Ethics Committee of the Ärztekammer Hamburg (PV6012, date of approval: 4 June 2019 and PV6012-4312_1-BO-ff, date of approval: 10 December 2021). The median age of the OvCA patient cohort was 61 years (range 32–86), and the median age of the healthy donors was 59 years (range 26–64) (Table S1). For detailed clinical data (e.g., FIGO stage, TNM status) of patients included in this study, see Table S2.

### 2.2. Preparation and Isolation of Peripheral Blood-, Malignant Ascites- and Tumor-Derived Mononuclear Cells

Mononuclear cells were isolated from the peripheral blood as previously described [29].

Malignant ascites was first centrifuged at 300× *g* for 5 min and resuspended in self prepared ACK lysing buffer (Ammonium-Chloride-Potassium Lysing Buffer, Thermo Fisher, Waltham, MA, USA). After a second centrifugation at 300× *g*, the cell pellets were washed with phosphate-buffered saline (PBS, Thermo Fisher), counted, and cryopreserved for FACS staining.

TILs were isolated from tumor tissue using a mix of Collagenases A, B, and D (Roche, Basel, Switzerland) and DNAse I (Thermo Fisher). In a 2 mL reaction tube, the digestion mix was added to finely minced tissue fragments and incubated for 1 h on a rotor at 37 °C. Afterwards, the digested tissue was slowly passed through a 40 µm filter and periodically rinsed with Hanks’ Balanced Salt Solution (HBSS, Thermo Fisher). The retrieved cell suspension was washed and centrifugated at 300× *g* for 5 min at 4 °C. ACK lysing buffer was added and incubated for 5 min at room temperature (RT). After washing the cells with PBS, they were counted and cryopreserved.

### 2.3. Multiparameter Flow Cytometry

Peripheral blood- (PB) and malignant ascites- (MA) derived mononuclear cells as well as TILs from patients with high-grade serous ovarian cancer and PB mononuclear cells from HDs were stained for multiparametric flow cytometry as previously described [29]. After FCR blocking (FcR blocking reagent, human, Miltenyi Biotec, Bergisch Gladbach, Germany), the cells were stained with the LIVE/DEAD^TM^ Fixable Near-IR dye (Thermo Fisher, Waltham, MA, USA) for exclusion of dead cells. Afterwards, surface staining was conducted with appropriate fluorochrome coupled surface antibodies (Supplementary Table S3, obtained from BioLegend, San Diego, CA, USA, or BD Biosciences, Franklin Lakes, NJ, USA). Subsequently, samples were fixed with 0.5% paraformaldehyde (Sigma Aldrich, St. Louis, MO, USA). All samples were read in on a BD FACSymphony A3 with FACS Diva version 8 (BD Biosciences).

### 2.4. T-Distributed Stochastic Neighbor Embedding Analyses

A subset of 3000 γδ TCR^+^ cells was randomly selected for single donors and merged into an individual expression matrix. As previously described [29], the following channels of our panel were excluded from the matrix to only include protein expression data of the molecules of interest in our tSNE analysis: viability, CD19, CD56, EpCAM, offset, residual, and time. Finally, 12,000 cells and 14 markers per tSNE group were used to create tSNE maps of the PB-, MA-, and TIL-derived γδ T cells. A perplexity parameter of 30 and iteration number of 550 was applied for the dimensionality reduction algorithm. The generated matrix consists of two columns corresponding to tSNE dimension 1 and dimension 2.

### 2.5. Statistical Analyses

FlowJo version 10.5.2. software (Treestar, Ashland, OR, USA) and Prism 7.0 software (GraphPad Software, San Diego, CA, USA) were used to analyze the data. Since our dataset was non-normally distributed, statistical analyses were performed by the Mann–Whitney test for two unpaired groups, or the Wilcoxon test for two paired groups. For bivariate correlation analysis, Pearson’s correlation and Spearman’s rank correlation coefficient were applied. Frequencies in the text were indicated as medians unless stated otherwise in the figure legend. *p*-values below 0.05 were considered significant, where *, **, ***, and **** indicate *p*-values between 0.01–0.05, 0.001–0.01, 0.0001–0.001, and <0.0001, respectively.

## 3. Results

### 3.1. γδ T Cells with a Vδ1 Chain Are Abundant in MALs and TILs

**Figure 1:**
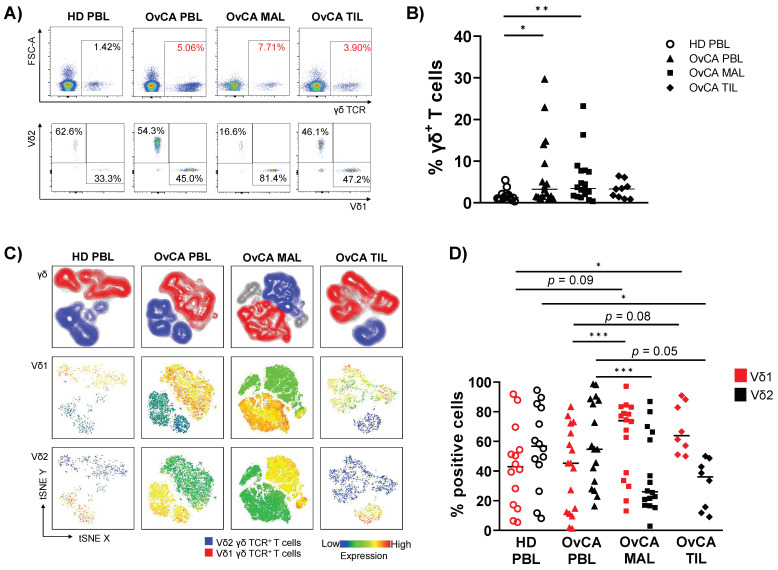
Enriched infiltration of Vδ1 T cells in malignant ascites- (MAL) and tumor-infiltrating lymphocytes (TIL) of ovarian cancer (OvCA) patients. Flow cytometric analysis regarding the co-expression of γδ TCR and the Vδ1 and Vδ2 receptor on CD3^+^ T cells was performed for peripheral blood- (PBL, triangles, *n* = 17), malignant ascites- (MAL, squares, *n* = 18), and tumor-infiltrating lymphocyte (TIL, diamonds, *n* = 9) samples from patients with high-grade serous ovarian cancer (OvCA), and PBL from healthy donors (HD, open circles, *n* = 14). (**A**) Representative flow cytometry data show the gating of γδ T cells (upper row); Vδ1 and Vδ2 T cells (lower row) for HD PBL (left); and OvCA PBL (second to left), MAL (second to right), and TIL (right) within CD3^+^ T cells. (**B**) Summary data depict the frequency of γδ T cells in HD PBL and OvCA PBL, MAL, and TIL. *p*-values were obtained by the Mann–Whitney test. * *p* < 0.05, ** *p* < 0.01. (**C**) T-distributed stochastic neighbor embedding (tSNE) analysis illustrates the distribution of Vδ1 and Vδ2 T cells within all γδ T cells in PBL from *n* = 4 HDs (left), and PBL (second to left), MAL (second to right), and TIL (right) from *n* = 4 OvCA patients, respectively. (**D**) Summary data show the frequency of Vδ1 (red) and Vδ2 (black) T cell subpopulations. Note that the number of samples for MAL and TIL was decreased to *n* = 16 and *n* = 8, respectively, as specimens with near to no Vδ1^+^ and Vδ2^+^ cells were excluded from this analysis. *p*-values were obtained by the Mann–Whitney test and Wilcoxon matched-pairs signed-rank test. * *p* < 0.05, *** *p* < 0.001.

To investigate T cell subsets in three different tumor-related compartments, we compared the frequency of γδ T cells, and the ratio of Vδ1 and Vδ2 T cells in PBLs, MALs, and TILs from patients with OvCA. In addition, PBLs were compared between patients with OvCA and age-matched HDs. For gating strategy, see Figure S1A and Figure 1A. The percentage of total γδ T cells within CD3^+^ T lymphocytes was significantly higher in PBLs and MALs from OvCA patients than in PBLs of HDs (*p* = 0.017, *p* = 0.005; Figure 1A,B). Some of the patients showed exceedingly high frequencies of up to 30% γδ T cells. The percentage of γδ T cells was positively correlated in OvCA patients in both PBLs with MALs as well as PBLs with TILs (*r* = 0.80 with *p* = 0.0002; and *r* = 0.73 with *p* = 0.03, respectively). No difference in percentages of γδ T cells was found between TILs and PBLs of HDs.

As illustrated in the t-distributed stochastic neighbor embedding (tSNE) analyses (Figure 1C), within the γδ T cell population, PBLs from OvCA patients and HDs displayed a similar ratio of Vδ1 and Vδ2 T cells, dominated by the Vδ2 T cell population. In contrast, the percentage of Vδ1 was markedly higher among MALs and TILs than PBLs (*p* = 0.0001, *p* = 0.078; Figure 1C,D). However, in the MALs two and in the TILs one sample were observed in which over 90% of all γδ T cells were neither Vδ1^+^ nor Vδ2^+^. Therefore, these samples were excluded from further analyses in which Vδ1 and Vδ2 subtypes were specifically characterized. As the homogeneity of the Vδ1^−^Vδ2^−^ cells was unclear, this subpopulation was not further analyzed.

Taken together, the inversed ratio of Vδ1 and Vδ2 cells in MALs and TILs compared to the PBLs suggests an increased migration of Vδ1 γδ T cells into the tumors directly, as well as into tissue in proximity to it.

### 3.2. Phenotypic Differentiation of γδ T Cells Exhibits Inter-Site Heterogeneity

**Figure 2:**
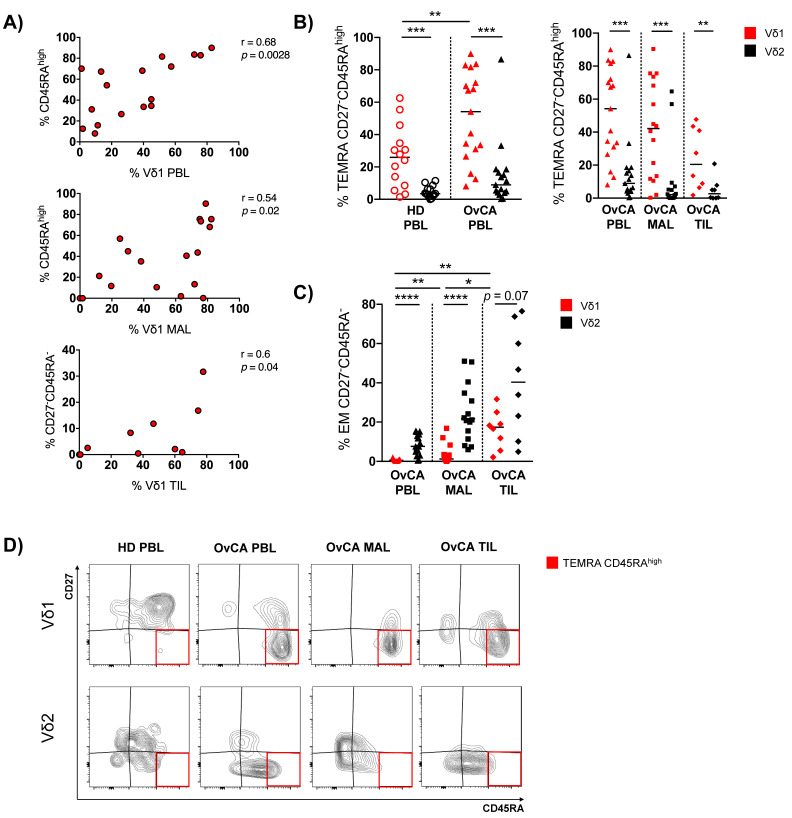
OvCA-derived Vδ1 T cells show a shift toward increased TEMRA and EM differentiation. γδ T cell differentiation was analyzed in Vδ1 (red) and Vδ2 (black) T cells via expression of CD45RA and CD27 for HD PBL (open circles, *n* = 14), and OvCA PBL (triangles, *n* = 17), MAL (squares, *n* = 16), and TIL (diamonds, *n* = 8). CD27^−^CD45RA^−^: effector memory (EM); CD27^−^CD45RA^high^: terminally differentiated (TEMRA) with high CD45RA expression. (**A**) Correlative analysis of the infiltration of Vδ1 T cells and the differentiation into CD27^−^CD45RA^high^ or CD27^−^CD45RA^−^ phenotypes, respectively, was performed for PBL (upper panel), MAL (middle panel), and TIL (lower panel) of OvCA patients. Pearson’s test was used to test for correlations. (**B**) Summary data depict the differentiation of Vδ1 vs. Vδ2 T cells into TEMRA CD27^−^CD45RA^high^ cells in HD PBL vs. OvCA PBL (left panel) vs. OvCA PBL vs. MAL vs. TIL (right panel). *p*-values were obtained by the Mann–Whitney test and Wilcoxon matched-pairs signed-rank test. ** *p* < 0.01, *** *p* < 0.001. (**C**) Summary data show the differentiation of Vδ1 vs. Vδ2 T cells into EM CD27^−^CD45RA^−^ cells in OvCA PBL vs. MAL vs. TIL. *p*-values were obtained by the Wilcoxon matched-pairs signed-rank test. * *p* < 0.05, ** *p* < 0.01, **** *p* < 0.0001. (**D**) Representative flow cytometry data show the differentiation of Vδ1 (upper panels) and Vδ2 (lower panels) T cells in HD PBL (left column), as well as PBL (second to left column), MAL (second to right column), and TIL (right column) from OvCA patients. The red squares indicate the gating of CD27^−^CD45RA^high^ cells.

We next assessed γδ T cell subtypes in distinct tumor-related compartments (TILs, MALs and PBLs). Vδ1 and Vδ2 T cells were separated into four different subpopulations based on the expression of CD27 and CD45RA. CD27^+^CD45RA^+^ = naïve (NA), CD27^+^CD45RA^−^ = central memory (CM), CD27^−^CD45RA^−^ = effector memory (EM), and CD27^−^CD45RA^+^ = terminally differentiated effector memory cells (TEMRA). In addition, we analyzed the frequency of a subset of TEMRA cells, the TEMRA CD45RA^high^ population (refer to Figure 2D for gating strategy). Functionally, TEMRA CD45RA^high^ cells are considered the most differentiated subset of human γδ T cells and have previously been described as dysfunctional with limited proliferation capacity [30]. For gating strategy, see Figure S1B.

Comparing HD PBLs with those of OvCA patients, Vδ1 γδ T cells showed a reduced fraction of NA cells (*p* = 0.009, Figure S3), whereas the TEMRA cells were increased among the Vδ1 PBLs of OvCA patients (*p* = 0.019; Figure S3). Interestingly, this maturation shift did not occur in the circulating Vδ2 PBLs of OvCA patients. Also, MAL- and TIL-derived Vδ1 cells of the patients showed higher levels of TEMRAs compared to their respective Vδ2 populations, but without reaching significance (Figure S3).

We further examined the CD45RA status (CD45RA^high^ vs. ^low^) within the TEMRAs of Vδ1 and Vδ2 cells. Differentiation into the TEMRA CD45RA^high^ phenotype was positively correlated with the prevalence of Vδ1 T cells in the PBLs and MALs (Figure 2A), but not in TILs from OvCA patients. In addition, this subset of TEMRA CD45RA^high^ cells was significantly increased in the Vδ1 PBLs from OvCA patients (but not within the Vδ2 subset) in comparison to Vδ1 PBLs of HDs (*p* = 0.009; Figure 2B). We also observed an increased fraction of these cells within the Vδ1 MALs and TILs, although this terminal differentiation status was less prominent in TILs in comparison to PBLs and MALs (Figure 2B). Instead, while the EM fraction was relatively low within the Vδ1 PBLs and MALs, their proportion was significantly higher in the TILs (Figure 2C). Furthermore, the differentiation into the EM CD27^−^CD45RA^−^ phenotype was positively correlated with infiltration of Vδ1 T cells in the TILs of OvCA patients (Figure 2A). In summary, the increased frequency of Vδ1 cells in OvCA had a variable phenotype depending on the different tissues.

### 3.3. Expression of Multiple Co-Regulatory Receptors by γδ T Cells Increases with Tumor Proximity

It has previously been shown that immune-exhaustion markers were highly expressed in tumor-infiltrating CD8^+^ T cells, representing a status of exhaustion with reduced production of effector cytokines and loss of the ability to eliminate cancer [31]. We therefore investigated the γδ T cell phenotype in greater detail in the different tissues. We compared the expression levels of co-regulatory receptors (CRRs), such as TIGIT, PD-1, TIM-3, Ox40, and the ectoenzymes CD39 and CD73 on all γδ, Vδ1, and Vδ2 T cells in PBLs, MALs, and TILs from OvCA patients and PBLs from HDs, respectively. The gating strategy can be assessed in Figures S1 and S2.

Summary data in Figure S4 show the distribution of CRRs in all γδ T cells. Here, TIGIT, PD-1, CD39, and Ox40 emerged as molecules of interest. TIGIT and PD-1 exhibited higher frequencies in all OvCA tissues (PBLs, MALs and TILs) in comparison to PBLs of HDs, whilst CD39 and Ox40 were detected exclusively in γδ TILs at an increased rate.

**Figure 3:**
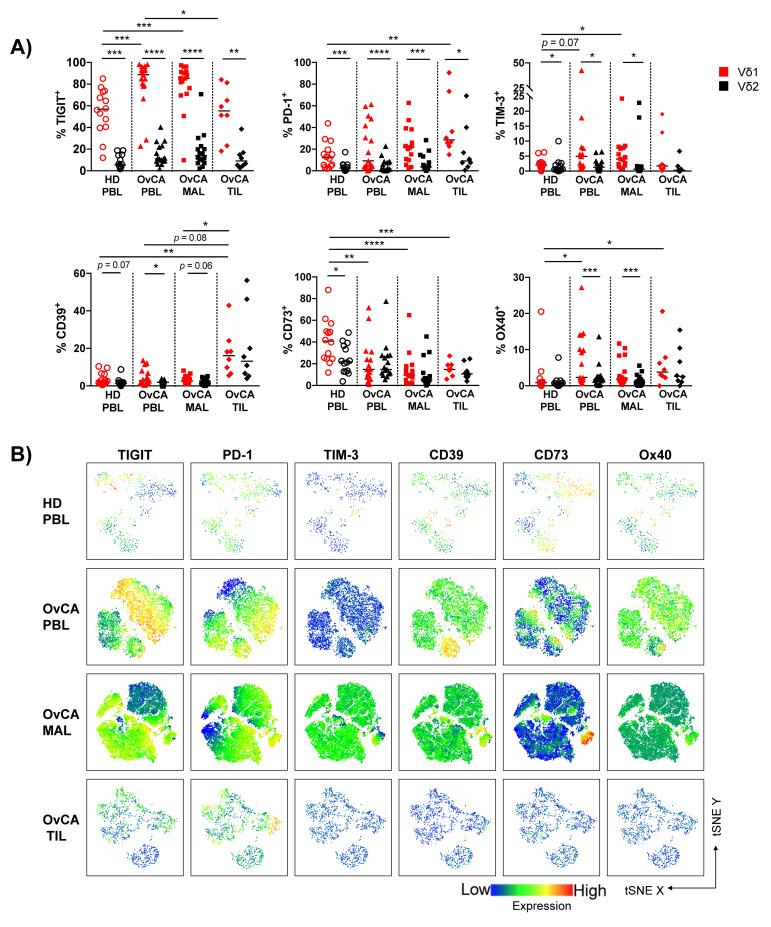
TIGIT, PD-1, CD39, and Ox40 emerge as co-regulatory receptors of interest in OvCA tissues and are preferentially expressed on Vδ1 T cells. The surface expression of TIGIT, PD-1, TIM-3, CD39, CD73, and Ox40 was compared between Vδ1 (red) and Vδ2 (black) T cells in HD PBL (open circles, *n* = 14), and OvCA PBL (triangles, *n* = 17), MAL (squares, *n* = 16), and TIL (diamonds, *n* = 8). (**A**) Summary data present the frequency of co-regulatory receptor (CRR)^+^ cells in Vδ1 and Vδ2 T cells. *p*-values were obtained by the Mann–Whitney test and Wilcoxon matched-pairs signed-rank test. * *p* < 0.05, ** *p* < 0.01, *** *p* < 0.001, **** *p* < 0.0001. (**B**) tSNE analysis illustrates the distribution of CRR^+^ T cells in the PBL from *n* = 4 HD (first row), and PBL (second row), MAL (third row) and TIL (fourth row) from *n* = 4 OvCA patients, respectively.

Hence, we further compared the CRR^+^ Vδ1 frequencies in HD PBLs with that in PBLs, MALs, and TILs of OvCA patients (Figure 3). We observed an increased proportion of TIGIT^+^, TIM-3^+^, and Ox40^+^ Vδ1 T cells in PBLs from OvCA patients compared to HD PBLs (*p* = 0.0004, *p* = 0.072, *p* = 0.010, respectively; Figure 3A). TIGIT^+^ and TIM-3^+^ cells were also more frequently expressed by Vδ1 in MALs than in PBLs of HDs (*p* = 0.0009, *p* = 0.038; Figure 3A). Finally, the TILs displayed an increased frequency of PD-1^+^, CD39^+^, and Ox40^+^ Vδ1 T cells in comparison to PBLs from HDs (*p* = 0.0064, *p* = 0.0004, *p* = 0.041, respectively; Figure 3A). The frequency of CD73^+^ Vδ1 T cells was reduced in each tissue of the OvCA patients in comparison to the HD PBLs (Figure 3A). The same CCR cluster occurred in the t-distributed stochastic neighbor embedding (tSNE) analyses (Figure 3B).

The comparison between the distribution of the CRRs expressed by Vδ1 vs. Vδ2 T cells within the particular OvCA compartments revealed that all of the analyzed checkpoints were preferentially expressed by Vδ1 and not Vδ2 γδ cells in PBLs, MALs, and TILs (Figure 3A and Figure S5).

Taken together, Vδ1 TILs displayed a characteristic proportion of PD-1^+^ and CD39^+^ cells, while TIGIT and TIM-3 showed the most dominant expression on Vδ1 PBLs and MALs from OvCA patients. These results indicate that the expression of TIGIT, PD-1, TIM-3, and CD39 on Vδ1 cells is dependent on interactions with the tumor microenvironment and differs between PBLs, MALs, and TILs in OvCA patients.

### 3.4. Vδ1 T Cells with Expression of TIGIT, PD-1 or CD39 Contained Subsets with More Differentiated Phenotypes

To better understand the differentiation of cells expressing co-regulatory molecules, we re-phenotyped the Vδ1 subsets with a positive expression of TIGIT, PD-1, and CD39 in regard to their maturation status.

**Figure 4:**
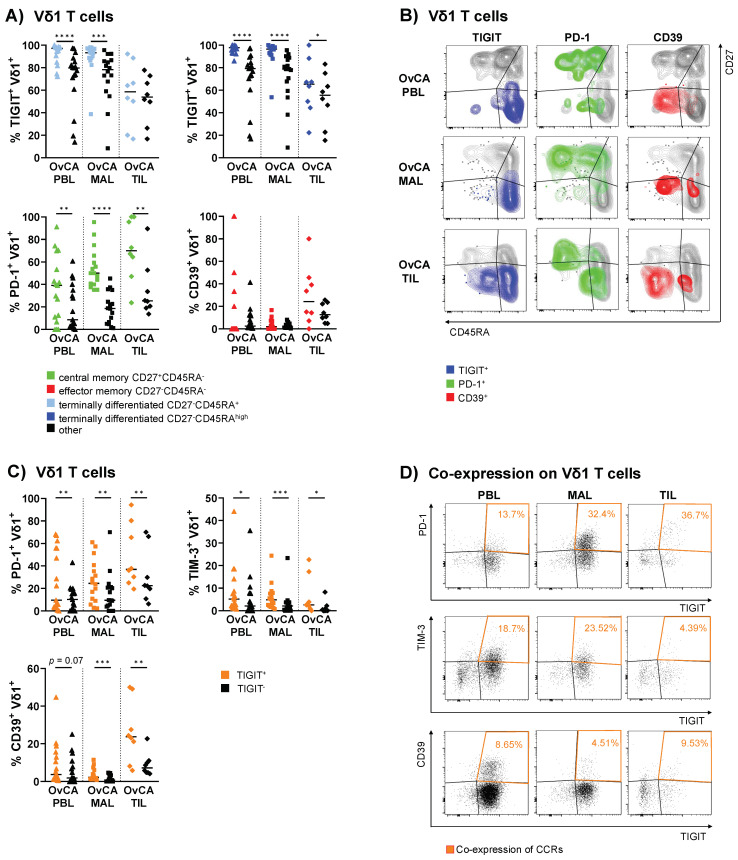
Co-regulatory molecules show specific clustering patterns on mature Vδ1 differentiation stages and are co-expressed in OvCA. The surface expression of TIGIT, PD-1, and CD39 on Vδ1 T cells was compared between differentiation stages based on CD27 and CD45RA expression in OvCA PBL (triangles, *n* = 17), MAL (squares, *n* = 16), and TIL (diamonds, *n* = 8). CD27^+^CD45RA^+^: naïve (NA); CD27^+^CD45RA^−^: central memory (CM); CD27^−^CD45RA^−^: effector memory (EM); CD27^−^CD45RA^+^: terminally differentiated effector memory (TEMRA); CD27^−^CD45RA^high^: TEMRA subpopulation with high CD45RA expression. (**A**) Summary data show the frequency of CRR^+^ cells in the respective differentiation stage vs. all other stages taken together. Upper row: TIGIT^+^ cells in all TEMRA (light blue, left panel) and TEMRA^high^ Vδ1 T cells (dark blue, right panel) vs. other (black; for TEMRA: NA, CM, and EM; for TEMRA^high^: NA, CM, EM, and TEMRA^low/int^). Lower row: PD-1^+^ cells in CM (green, left panel) and CD39^+^ cells in EM Vδ1 T cells (red, right panel) vs. other (black; for CM: NA, EM, and TEMRA; for EM: NA, CM, and TEMRA). *p*-values were obtained by the Wilcoxon matched-pairs signed-rank test. * *p* < 0.05, ** *p* < 0.01, *** *p* < 0.001, **** *p* < 0.0001. (**B**) Representative flow cytometry data illustrate the expression of TIGIT (left column), PD-1 (middle column), and CD39 (right column) in Vδ1 T cells based on the expression of CD27 and CD45RA in OvCA PBL (first row), MAL (second row), and TIL (third row), respectively. (**C**) Summary data depict the expression of PD-1, TIM-3, and CD39 on TIGIT^+^ (orange) vs. TIGIT^−^ (black) cells on Vδ1 T cells. *p*-values were obtained by the Wilcoxon matched-pairs signed-rank test. * *p* < 0.05, ** *p* < 0.01, *** *p* < 0.001. (**D**) Representative flow cytometry data illustrate the (co-)expression of TIGIT and PD-1 (first row), TIM-3 (second row), and CD39 (third row) in Vδ1 T cells in OvCA PBL (left column), MAL (middle column), and TIL (right column), respectively.

Within PBLs and MALs from OvCA patients, expression of TIGIT was a specific feature of TEMRA cells, in particular of the TEMRA CD45RA^high^ Vδ1 cells (TIGIT^+^ TEMRA CD45RA^high^ vs. TIGIT^+^ non TEMRA CD45RA^high^ cells (defined as “other” = NA, CM, EM, and TEMRA CD45RA^low/int^ Vδ1 T cells): *p* < 0.0001 and *p* < 0.0001, respectively; Figure 4A,B).

Here, the detected expression of TIGIT reached extraordinarily high levels of up to 100%. Conversely, PD-1^+^ Vδ1 cells displayed a less mature phenotype. These cells were mainly contained in the CM cells of the PBLs, MALs, and the majority among the TILs (*p* = 0.008; Figure 4A,B). Meanwhile, CD39^+^ Vδ1 T cells were primarily observed within EM TILs, although this difference was not significant (*p* = 0.148; Figure 4A,B). For TIM-3 and Ox40, no clear clustering was asserted at specific maturation stages (Figures S6 and S7). CD73 was mainly expressed on NA cells (Figure S7). Additional CRR expression data on the analyzed differentiation stages for Vδ1 and, moreover, Vδ2 T cells is shown in Figures S7 and S8, respectively. For further visualization, the localization of the CCR^+^ cells is depicted within the four differentiation stages of Vδ1 T cells (Figure 4B).

To summarize, our data showed that the expression of TIGIT, PD-1, and CD39 was generally associated with specific maturation stages of the Vδ1 T cells in OvCA.

### 3.5. PD-1 and CD39 Are More Frequently Co-Expressed with TIGIT on Vδ1 T Cells Isolated from PBLs, MALs and TILs

Previous results have indicated that co-expression of PD-1 with either TIM-3 or LAG-3 (lymphocyte-activation gene 3) on T cells is associated with immune exhaustion in patients with solid cancers [31]. In this investigation, we showed the expression of these checkpoints on different phenotypic Vδ1 cells in OvCA for the first time. In addition, we found an accumulation of CCR^+^ cells in both the EM and TEMRA compartment, indicating potential co-expression (Figure 4B). Therefore, we further assessed co-expression of the CCRs in PBLs, MALs, and TILs from OvCA patients and PBLs from HDs.

In the following section, only the significant co-expression patterns observed in this study are discussed (for additional data regarding co-expression, see Figure S9). Since TIGIT expression was the most prevalent compared to other CRRs among Vδ1 cells in OvCA, the comparison of TIGIT^+^ vs. TIGIT^−^ Vδ1 T cell subsets revealed a significantly increased frequency of PD-1^+^ cells within the TIGIT^+^ Vδ1 T cells. This co-expression was observed in the PBLs (*p* = 0.005), MALs (*p* = 0.009) and TILs (*p* = 0.008; Figure 4C,D). The percentage of co-expressing cells increased with tumor proximity (Figure 4C,D). Also, TIM-3 was more frequently expressed by TIGIT^+^ Vδ1 T cells than by TIGIT^−^ Vδ1 cells from PBLs (*p* = 0.013), MALs (*p* = 0.0003), and TILs (*p* = 0.016; Figure 4C), however, the frequency decreased with tumor proximity (Figure 4C,D). In addition, we observed a similar pattern for CD39, which was co-expressed by TIGIT^+^ vs. TIGIT^−^ Vδ1 cells (PBL *p* = 0.065, MAL *p* = 0.0003, TIL *p* = 0.008) with the highest frequency of CD39^+^TIGIT^+^ Vδ1 T cells found in the TILs (Figure 4C,D). Interestingly, no co-expression with the costimulatory receptor Ox40 was found in these Vδ1 subsets.

Overall, there was a high inter-patient variability in the distributions of the double-positive cells. However, the mean frequencies of double-positive cells showed a characteristic increase of PD-1^+^ or CD39^+^ Vδ1 T cells co-expressing TIGIT from PBL to MAL to TIL.

## 4. Discussion

Our study provides a phenotypic characterization of matched PB-, MA-, and tumor-derived γδ T cells in OvCA patients. We observed an increased proportion of Vδ1 T cells in MALs and TILs in OvCA, whereas Vδ2 T cells made up the majority in the PBLs of both OvCA patients and HDs. The Vδ1 cells in MALs showed an increased number of cells carrying a TEMRA phenotype with an aberrant subpopulation of CD27^−^CD45RA^high^ Vδ1 T cells. In contrast, the increased EM differentiation was most prominent in the TILs. In all three tissues from patients with OvCA, there appeared an increased frequency of co-regulatory receptor (CRR)^+^ cells in the Vδ1 population. However, there were clear site-dependent differences in the expression of individual CRRs on the Vδ1 cells from OvCA patients. PBLs exhibited an increased frequency of TIGIT^+^, TIM-3^+^, and Ox40^+^ T cells. MALs only showed an increased frequency of TIGIT^+^ and TIM-3^+^ cells, whereas TILs characteristically had higher frequencies of PD-1^+^, CD39^+^, and Ox40^+^ cells in comparison to Vδ1 cells from HD PBLs. In contrast, all of the γδ T cells exhibited a reduced fraction of CD73^+^ cells. The upregulated expression of co-inhibitory receptors in Vδ1 T cells in OvCA could be assigned to particular differentiation stages; whereas CD73 was found predominantly in NA cells, PD-1 expression was related to the CMs and the bulk of CD39^+^ Vδ1 cells were EMs within the TILs. Strikingly, the TEMRA and TEMRA CD27^−^CD45RA^high^ subpopulation showed extraordinarily high frequencies of TIGIT up to 100%. Finally, despite the association between checkpoint expression and differentiation stage, increased co-expression of PD-1, TIM-3, and CD39 with TIGIT was detected in the total of Vδ1 γδ T cells in every tissue site in OvCA, especially EM and TEMRA. These findings altogether imply an increased state of exhaustion in Vδ1 T cells in OvCA.

Regarding γδ cells, in our cohort, throughout different tissues the percentage of γδ T cells was about 3% per total CD3^+^ T cells. Exceptions to this were a subgroup of PBLs and MALs with proportions of up to 30% γδ T cells. Unfortunately, we did not have matched TIL samples for these patients, so it remains unclear whether the γδ frequencies in the respective TIL population would have been elevated as well. Although we were unable to detect the elevated frequencies for γδ TILs, it is arguable that this might be due to the hitherto low number of samples and that we might find higher frequencies in larger studies. There have not yet been extensive studies about the infiltration of γδ T cells in different OvCA compartments. In line with our data, Foord et al. showed similar distributions of γδ T cells in PBLs, MALs, and TILs in OvCA [32]. Even though according to our findings, the level of γδ TILs did not seem to be generally increased in OvCA TILs compared to PBLs or MALs, tumor tissue enriched in γδ T cells was found to correlate positively with an advanced disease (classified by a higher FIGO stage), larger tumor size, and lymph node metastasis [33].

Concordant with other studies, we found that for MALs and TILs from OvCA patients, the infiltration of tissue-resident Vδ1 T cells was higher than that of Vδ2 T cells in comparison to the Vδ1/Vδ2 distribution within the PBLs from OvCA patients or HDs [33]. This increased Vδ1 infiltration into tumor tissue has recently been shown in multiple solid cancers, including colorectal cancer, melanoma, and non-small cell lung cancer [8,33]. The increased ratio of Vδ1/Vδ2 T cells also seems to have prognostic relevance. Chen et al. found an association of increased Vδ1 infiltration in OvCA tissues with advanced clinical FIGO stage and lymph node metastasis. This indicates a critical role of this γδ population in cancer progression and invasiveness for OvCA [33]. In line with this, Cordova et al. identified increased levels of Vδ2 T cells in melanoma to correlate with an early stage of disease and the absence of metastasis [34]. Additionally, in colorectal cancer patients, the abundance of Vδ2 T cells correlated with a longer 5-year disease free survival rate [35].

To our knowledge, our study is the first phenotypic analysis including a broad panel of immune checkpoint molecules in OvCA. However, there are some observations for other tumor entities analyzing the differentiation status of Vδ1 and Vδ2 T cells. Fisher et al. described Vδ1 T cells in neuroblastoma as less differentiated than Vδ2 T cells, showing higher numbers of CM and NA cells, whereas the EM fraction was decreased [36]. The TEMRA and TEMRA CD27^−^CD45RA^high^ compartment, however, was not analyzed in this study. Concordant with our results, for squamous skin cancer, Presti et al. described both Vδ1 and Vδ2 T cells in TILs as bearing mainly the EM phenotype, whereas in PB from the same patients, the TEMRA phenotype was predominant [37]. Similar to αβ T cells, there seems to be a variability in γδ differentiation depending on the tumor entity, suggesting a variable role of γδ subsets that might induce pro- or antitumoral effects. Our examination of the differentiation status also showed a shift toward a dominant TEMRA fraction in Vδ1 PBLs and MALs, especially of TEMRA CD27^−^CD45RA^high^ cells. This population has not yet found much recognition in phenotypic analyses. It was first described for PBLs by Odaira et al. as a subset with the greatest lack of expansion and proliferation capacity [30]. This indicates a more severe status of exhaustion in these CD45RA^high^ -expressing cells in contrast to CD45RA^low/int^ TEMRA cells. To our knowledge, there is yet no description of the distribution of these cells in different tissues, not to mention in different malignancies. Interestingly, we found mainly the Vδ1 subpopulation to bear the CD27^−^CD45RA^high^ phenotype. In line with this, we recently published data showing a similar pattern of this terminal differentiation in Vδ1 T cells in acute myeloid leukemia (AML) and multiple myeloma (MM) [29].

Regarding the expression of co-inhibitory checkpoint molecules, we found an increased percentage of TIGIT^+^, PD-1^+^, and TIM-3^+^ cells within the Vδ1 cells compared to the Vδ2 cells. This pattern was observed in the majority of patients across all three tissue compartments in OvCA. In contrast, expression of CD39 and CD73 showed similar levels within Vδ1 and Vδ2 cells in OvCA. Additionally, TIGIT^+^, PD-1^+^, TIM-3^+^, CD39^+^, and Ox40^+^ cells were more frequent within the Vδ1 subset in tissues from OvCA patients than in HDs. Thus, checkpoint expression on Vδ1 cells exclusively separated OvCA subjects from HDs. Again, we previously found a very similar immunologic signature in bone marrow-derived γδ T cells from patients with AML and MM [29]. In line with our data, a comparable checkpoint signature was also demonstrated in the blood of HIV patients. In a panel of checkpoints, a significantly increased frequency of TIGIT^+^ and TIM-3^+^ γδ cells was found in HIV patients compared to controls [38].

There have not yet been other publications comparing the expression of immune checkpoints on OvCA Vδ1 vs. Vδ2 γδ cells or vs. Vδ1 HDs. Among the CRRs analyzed, TIGIT was the most frequently expressed one. Moreover, we observed an association between expression of TIGIT and TEMRA CD45RA^high^ Vδ1 cells. This combination might point to a link between terminal differentiation and expression of co-inhibitory receptors, resulting in functional exhaustion. It has been shown that TIGIT negatively regulates cytotoxic effector cell functions and clinical trials are already testing the advantages of TIGIT blockade in different tumor entities [39]. Notably, our study shows that TIGIT^+^ cells were increased within the PBLs and MALs in OvCA but not within the TILs. This finding was further supported by the different phenotype of γδ populations we observed in the TILs. Instead of CD45RA^high^ cells, EM Vδ1 cells represented a dominant γδ population in tumor tissue. These results, however, must be evaluated with caution due to the low number of TIL specimens. Generally, there are a few comparable studies demonstrating an increased proportion of TIGIT^+^CD8^+^ T cells within tumor tissue [40].

Our study revealed that PD-1 and CD39 were characteristically expressed by Vδ1 TILs, which displayed the EM phenotype in a high frequency. Mohme et al. showed a similar characteristic elevation of PD-1 and CD39 on CD8^+^ TILs in glioblastoma [41]. Regarding the functionality of checkpoints in γδ T cells, in primary γδ T cells, PD-1 expression was rapidly induced upon antigenic stimulation, whereby IFN-γ production in responses were lower in PD-1^+^ than in PD-1^−^ γδ T cells [42,43]. Increased expression of CD39 in combination with CD103 has already been described as a phenotypic signature for tumor antigen-specific CD8^+^ T cells [24,44], hypothesizing that γδ T cells in the tumor carry a similar immune signature as CD8^+^ T cells. Hu et al. showed that γδ T cells expressing CD39 had a stronger immunosuppressive activity than regulatory CD4^+^ or CD8^+^ T cells acting via the adenosine-induced pathway instead of TGF-β or IL-10 [45]. Our studies also showed an increased expression of TIM-3 on Vδ1 T cells, although the cell frequencies were significantly lower. Consistent with our findings, Xioma et al. also found an increased frequency of TIM-3^+^ cells within the total γδ T cell population in colorectal carcinoma. Moreover, anti-TIM-3 treatment enhanced the cytotoxicity of Vδ2 T cells in vitro [46]. In addition, in breast cancer, the additional treatment of a TIM-3 inhibitor together with a bispecific T-cell engager directed against CD3 and EpCAM further enhanced the anti-tumor toxicity of γδ T cells [47].

With regard to the increased co-expression of PD-1, TIM-3, and CD39 with TIGIT, we were unable to find comparable analyses. However, there are publications showing co-expression of those markers on CD8^+^ T cells [23,26,48]. TIGIT^+^CD8^+^ TILs co-expressing PD-1 or PD-1^+^TIM-3^+^ T cells have been described as highly dysfunctional [49,50,51]. When TIGIT and PD-1 were blocked in combination, this additively increased proliferation, cytokine production, and degranulation of these T cells [49,52].

So far, despite the higher cytotoxicity of Vδ1 cells, Vδ2 cells have been the focus of γδ-based immunotherapeutic strategies due to their better cultivability. Our data show a higher infiltration of Vδ1 cells in tumor tissue, as well as a distinct immune signature on Vδ1 in contrast to Vδ2 cells. Even though our patient number was still small, our analyses clearly showed an increased frequency of TIGIT^+^, PD-1^+^, and CD39^+^ γδ cells in the tissues of OvCA patients. Identifying TIGIT in combination with PD-1 and CD39 as targets of interest for Vδ1 T cells, this raises the question whether inhibition of these receptors by immunomodulatory treatment might reinvigorate cytotoxic Vδ1 T cells in OvCA. Due to their additional interaction possibility by co-regulatory receptors including NKRs, balance between inhibitory and activating receptor signaling could be particularly important for activation of γδ T cells and their immune surveillance potential.

## Data Availability

The datasets used and/or analyzed during the current study are available from the corresponding authors on reasonable request (f.brauneck@uke.de).

## Supplementary Materials

The following supporting information can be downloaded at: https://www.mdpi.com/article/10.3390/cells11060964/s1, Table S1: Patient characteristics of patients included in the study; Table S2: Clinical characteristics of patients included in the study; Table S3: Antibodies and clones used in the study; Figure S1: Flow cytometry gating strategy to identify (Vδ1 and Vδ2) γδ T cells; Figure S2: Fluorescence minus one (FMO) flow cytometry data to identify positive expression; Figure S3: Differentiation into phenotypes; Figure S4: Expression of co-inhibitory receptors, ectonucleotidases, and activation markers on γδ T cells; Figure S5: Expression of co-inhibitory receptors, ectonucleotidases, and activation markers on Vδ1 and Vδ2 T cells; Figure S6: Expression of co-inhibitory receptors, ectonucleotidases, and activation markers on Vδ1 CD27^−^CD45RA^high^ T cells; Figure S7: Expression of co-inhibitory receptors, ectonucleotidases, and activation markers on Vδ1 differentiation stages; Figure S8: Expression of co-inhibitory receptors, ectonucleotidases, and activation markers on Vδ2 differentiation stages; Figure S9: Co-expression of co-inhibitory receptors, ectonucleotidases, and activation markers on Vδ1 T cells.

## Abbreviations

AML	Acute myeloid leukemia
ATP	Adenosine-triphosphate
BiTE	Bispecific T cell engager
CAR-T cell	Chimeric antigen receptor T cell;
CCR	Co-regulatory receptor
CD27	Tumor necrosis factor receptors superfamily member 7
CD39	Ectonucleoside triphosphate diphosphohydrolase-1
CD45RA	Protein tyrosine phosphatase, receptor type C
CD73	Ecto-5′-nucleotidase
CD103	alpha E integrin
CM	Central memory
EM	Effector memory
EpCAM	Epithelial cell adhesion molecule
FIGO	International Federation of Gynecology and Obstetrics
FMO	Fluorescence minus one
GM-CSF	Granulocyte-macrophage colony-stimulating factor
HBSS	Hanks’ balanced salt solution
HD	Healthy donor
HIV	Human Immunodeficiency Virus
HLA	Human leukocyte antigen
IFN-γ	Interferon-gamma
IL	Interleukin
LAG-3	Lymphocyte activation gene 3
MA	Malignant ascites
MAL	Malignant ascites lymphocyte
MDSC	Myeloid-derived suppressor cell
MFC	Multiparameter flow cytometry
MFI	Median fluorescence intensity
MHC	Major histocompatibility complex
MM	Multiple myeloma
NA	Naïve
NKG2D	Natural killer group 2D
NKR	Natural killer receptor
OvCA	Ovarian cancer
Ox40	Tumor necrosis factor receptors superfamily member 40
PB	Peripheral blood
PBL	Peripheral blood lymphocyte
PBMC	Peripheral blood mononuclear cell
PBS	Phosphate buffered saline
PD-1	Programmed cell death-1
RBC	Red blood cell
RT	Room temperature
SEER	Surveillance, Epidemiology, and End Results Program
TCR	T cell receptor
TEMRA	Terminally differentiated effector memory
TGF-β	Transforming growth factor beta;
TIGIT	T cell immunoreceptor with Ig and ITIM domains
TIL	Tumor-infiltrating lymphocyte
TIM-3	T cell immunoglobulin and mucin domain-containing protein 3
TNF-α	Tumor necrosis factor alpha
tSNE	t-distributed stochastic neighbor embedding

## References

[1] Charkhchi P., Cybulski C., Gronwald J., Wong F.O., Narod S.A., Akbari M.R. (2020). CA125 and Ovarian Cancer: A Comprehensive Review. Cancers.

[2] Goff B.A., Mandel L.S., Drescher C.W., Urban N., Gough S., Schurman K.M., Patras J., Mahony B.S., Andersen M.R. (2007). Development of an ovarian cancer symptom index: Possibilities for earlier detection. Cancer.

[3] Webb P.M., Jordan S.J. (2017). Epidemiology of epithelial ovarian cancer. Best Pract. Res. Clin. Obstet. Gynaecol..

[4] Correia D.V., Lopes A.C., Silva-Santos B. (2013). Tumor cell recognition by γδ T lymphocytes. Oncoimmunology.

[5] Simões A.E., Di Lorenzo B., Silva-Santos B. (2018). Molecular determinants of target cell recognition by human γδ T cells. Front. Immunol..

[6] Kabelitz D., Serrano R., Kouakanou L., Peters C., Kalyan S. (2020). Cancer immunotherapy with γδ T cells: Many paths ahead of us. Cell. Mol. Immunol..

[7] Deseke M., Prinz I. (2020). Ligand recognition by the γδ TCR and discrimination between homeostasis and stress conditions. Cell. Mol. Immunol..

[8] Wesch D., Kabelitz D., Oberg H.H. (2020). Tumor resistance mechanisms and their consequences on γδ T cell activation. Immunol. Rev..

[9] Park J.H., Lee H.K. (2021). Function of γδ T cells in tumor immunology and their application to cancer therapy. Exp. Mol. Med..

[10] Hayday A.C. (2019). γδ T Cell Update: Adaptate Orchestrators of Immune Surveillance. J. Immunol..

[11] Kabelitz D., Kalyan S., Oberg H.H., Wesch D. (2013). Human vδ2 versus non-vδ2 γδ t cells in antitumor immunity. Oncoimmunology.

[12] Khairallah C., Chu T.H., Sheridan B.S. (2018). Tissue Adaptations of Memory and Tissue-Resident Gamma Delta T Cells. Front. Immunol..

[13] Gertner-Dardenne J., Castellano R., Mamessier E., Garbit S., Kochbati E., Etienne A., Charbonnier A., Collette Y., Vey N., Olive D. (2012). Human Vγ9Vδ2 T Cells Specifically Recognize and Kill Acute Myeloid Leukemic Blasts. J. Immunol..

[14] Poggi A., Zocchi M.R. (2014). γδ T lymphocytes as a first line of immune defense: Old and new ways of antigen recognition and implications for cancer immunotherapy. Front. Immunol..

[15] Zhao Y., Niu C., Cui J. (2018). Gamma-delta (γδ) T Cells: Friend or Foe in Cancer Development. J. Transl. Med..

[16] Wu P., Wu D., Ni C., Ye J., Chen W., Hu G., Wang Z., Wang C., Zhang Z., Xia W. (2014). γδT17 cells promote the accumulation and expansion of myeloid-derived suppressor cells in human colorectal cancer. Immunity.

[17] Chauvin J.M., Zarour H.M. (2020). TIGIT in cancer immunotherapy. J. Immunother. Cancer.

[18] Simon S., Labarriere N. (2018). PD-1 expression on tumor-specific T cells: Friend or foe for immunotherapy?. Oncoimmunology.

[19] Wolf Y., Anderson A.C., Kuchroo V.K. (2020). TIM3 comes of age as an inhibitory receptor. Nat. Rev. Immunol..

[20] Anderson A.C., Joller N., Kuchroo V.K. (2016). Lag-3, Tim-3, and TIGIT: Co-inhibitory Receptors with Specialized Functions in Immune Regulation. Immunity, NIH Public Access 44, 989–1004.ag-3, Tim-3, and TIGIT: Co-inhibitory Receptors with Specia. Immunity.

[21] Pauken K.E., Wherry E.J. (2015). Overcoming T cell exhaustion in infection and cancer. Trends Immunol..

[22] Sun G., Sun X., Li W., Liu K., Tian D., Dong Y., Sun X., Xu H., Zhang D. (2018). Critical role of OX40 in the expansion and survival of CD4 T-cell-derived double-negative T cells. Cell Death Dis..

[23] Canale F.P., Ramello M.C., Núñez N., Furlan C.L.A., Bossio S.N., Serrán M.G., Boari J.T., Del Castillo A., Ledesma M., Sedlik C. (2018). CD39 expression defines cell exhaustion in tumor-infiltrating CD8+ T cells. Cancer Res..

[24] Duhen T., Duhen R., Montler R., Moses J., Moudgil T., De Miranda N.F., Goodall C.P., Blair T.C., Fox B.A., McDermott J.E. (2018). Co-expression of CD39 and CD103 identifies tumor-reactive CD8 T cells in human solid tumors. Nat. Commun..

[25] Roh M., Wainwright D.A., Wu J.D., Wan Y., Zhang B. (2020). Targeting CD73 to augment cancer immunotherapy. Curr. Opin. Pharmacol..

[26] Brauneck F., Haag F., Woost R., Wildner N., Tolosa E., Rissiek A., Vohwinkel G., Wellbrock J., Bokemeyer C., Wiesch J.S. (2021). Increased frequency of TIGIT CD73-CD8 T cells with a TOX TCF-1low profile in patients with newly diagnosed and relapsed AML. Oncoimmunology.

[27] Turcotte M., Spring K., Pommey S., Chouinard G., Cousineau I., George J., Chen G.M., Gendoo D.M.A., Haibe-Kains B., Karn T. (2015). CD73 is associated with poor prognosis in high-grade serous ovarian cancer. Cancer Res..

[28] Allard B., Allard D., Buisseret L., Stagg J. (2020). The adenosine pathway in immuno-oncology. Nat. Rev. Clin. Oncol..

[29] Brauneck F., Weimer P., Schulze zur Wiesch J., Weisel K., Leypoldt L., Vohwinkel G., Fritzsche B., Bokemeyer C., Wellbrock J., Fiedler W. (2021). Bone Marrow-Resident Vδ1 T Cells Co-express TIGIT With PD-1, TIM-3 or CD39 in AML and Myeloma. Front. Med..

[30] Odaira K., Kimura S., Fujieda N., Kobayashi Y., Kambara K., Takahashi T., Izumi T., Matsushita H., Kakimi K. (2016). CD27-CD45+ γδ T cells can be divided into two populations, CD27-CD45int and CD27-CD45hi with little proliferation potential. Biochem. Biophys. Res. Commun..

[31] Wherry E.J., Kurachi M. (2015). Molecular and cellular insights into T cell exhaustion. Nat. Rev. Immunol..

[32] Foord E., Arruda L.C.M., Gaballa A., Klynning C., Uhlin M. (2021). Characterization of ascites- and tumor-infiltrating γδ T cells reveals distinct repertoires and a beneficial role in ovarian cancer. Sci. Transl. Med..

[33] Chen X., Shang W., Xu R., Wu M., Zhang X., Huang P., Wang F., Pan S. (2019). Distribution and functions of γδ T cells infiltrated in the ovarian cancer microenvironment. J. Transl. Med..

[34] Cordova A., Toia F., la Mendola C., Orlando V., Meraviglia S., Rinaldi G., Todaro M., Cicero G., Zichichi L., Donni P.L. (2012). Characterization of Human γδ T Lymphocytes Infiltrating Primary Malignant Melanomas. PLoS ONE.

[35] Meraviglia S., Lo Presti E., Tosolini M., La Mendola C., Orlando V., Todaro M., Catalano V., Stassi G., Cicero G., Vieni S. (2017). Distinctive features of tumor-infiltrating γδ T lymphocytes in human colorectal cancer. Oncoimmunology.

[36] Fisher J.P.H., Yan M., Heuijerjans J., Carter L., Abolhassani A., Frosch J., Wallace R., Flutter B., Capsomidis A., Hubank M. (2014). Neuroblastoma killing properties of Vδ2 and Vδ2-negative γδT cells following expansion by artificial antigen-presenting cells. Clin. Cancer Res..

[37] Lo Presti E., Toia F., Oieni S., Buccheri S., Turdo A., Mangiapane L.R., Campisi G., Caputo V., Todaro M., Stassi G. (2017). Squamous cell tumors recruit γδ T cells producing either IL17 or IFNγ depending on the tumor stage. Cancer Immunol. Res..

[38] Belkina A.C., Starchenko A., Drake K.A., Proctor E.A., Pihl R.M.F., Olson A., Lauffenburger D.A., Lin N., Snyder-Cappione J.E. (2018). Multivariate Computational Analysis of Gamma Delta T Cell Inhibitory Receptor Signatures Reveals the Divergence of Healthy and ART-Suppressed HIV+ Aging. Front. Immunol..

[39] Rotte A., Sahasranaman S., Budha N. (2021). Targeting tigit for immunotherapy of cancer: Update on clinical development. Biomedicines.

[40] Woroniecka K., Chongsathidkiet P., Rhodin K., Kemeny H., Dechant C., Harrison Farber S., Elsamadicy A.A., Cui X., Koyama S., Jackson C. (2018). T-cell exhaustion signatures vary with tumor type and are severe in glioblastoma. Clin. Cancer Res..

[41] Mohme M., Schliffke S., Maire C.L., Runger A., Glau L., Mende K.C., Matschke J., Gehbauer C., Akyuz N., Zapf S. (2018). Immunophenotyping of Newly Diagnosed and Recurrent Glioblastoma Defines Distinct Immune Exhaustion Profiles in Peripheral and Tumor-infiltrating Lymphocytes. Clin. Cancer Res..

[42] Iwasaki M., Tanaka Y., Kobayashi H., Murata-Hirai K., Miyabe H., Sugie T., Toi M., Minato N. (2011). Expression and function of PD-1 in human γδ T cells that recognize phosphoantigens. Eur. J. Immunol..

[43] Hoeres T., Holzmann E., Smetak M., Birkmann J., Wilhelm M. (2019). PD-1 signaling modulates interferon-γ production by Gamma Delta (γδ) T-Cells in response to leukemia. Oncoimmunology.

[44] Eiva M.A., Omran D.K., Chacon J.A., FPowell D.J. (2022). Systhematic analysis of CD39, CD103, CD137, and PD-1 as biomarkers for naturally occuring tumor antigen-specific TILs. Eur J Immunol..

[45] Hu G., Wu P., Cheng P., Zhang Z., Wang Z., Yu X., Shao X., Wu D., Ye J., Zhang T. (2017). Tumor-infiltrating CD39+ γδTregs are novel immunosuppressive T cells in human colorectal cancer. Oncoimmunology.

[46] Li X., Lu H., Gu Y., Zhang X., Zhang G., Shi T., Chen W. (2020). Tim-3 suppresses the killing effect of Vγ9Vδ2 T cells on colon cancer cells by reducing perforin and granzyme B expression. Exp. Cell Res..

[47] Guo Q., Zhao P., Zhang Z., Zhang J., Zhang Z., Hua Y., Han B., Li N., Zhao X., Hou L. (2020). TIM-3 blockade combined with bispecific antibody MT110 enhances the anti-tumor effect of γδ T cells. Cancer Immunol. Immunother..

[48] Ma J., Zheng B., Goswami S., Meng L., Zhang D., Cao C., Li T., Zhu F., Ma L., Zhang Z. (2019). PD1Hi CD8+ T cells correlate with exhausted signature and poor clinical outcome in hepatocellular carcinoma. J. Immunother. Cancer.

[49] Chauvin J.M., Pagliano O., Fourcade J., Sun Z., Wang H., Sander C., Kirkwood J.M., Chen T.H.T., Maurer M., Korman A.J. (2015). TIGIT and PD-1 impair tumor antigen-specific CD8+ T cells in melanoma patients. J. Clin. Investig..

[50] Roussel M., Le K.S., Granier C., Gutierrez F.L., Foucher E., Le Gallou S., Pangault C., Xerri L., Launay V., Lamy T. (2021). Functional characterization of PD11TIM31 tumor-infiltrating T cells in DLBCL and effects of PD1 or TIM3 blockade. Blood Adv..

[51] Li X., Wang R., Fan P., Yao X., Qin L., Peng Y., Ma M., Asley N., Chang X., Feng Y. (2019). A comprehensive analysis of key immune checkpoint receptors on tumor-infiltrating t cells from multiple types of cancer. Front. Oncol..

[52] Ge Z., Zhou G., Campos Carrascosa L., Gausvik E., Boor P.P.C., Noordam L., Doukas M., Polak W.G., Terkivatan T., Pan Q. (2021). TIGIT and PD1 Co-blockade Restores ex vivo Functions of Human Tumor-Infiltrating CD8+ T Cells in Hepatocellular Carcinoma. CMGH.

