# Supplementary material for: Tissue-Specific Expression of TIGIT, PD-1, TIM-3, and CD39 by γδ T Cells in Ovarian Cancer
Source: Cells. 2022 Mar 11;11(6):964. doi: 10.3390/cells11060964 (PMC8946192; doi:10.3390/cells11060964)
Supplement: Supplementary file 1 [file cells-11-00964-s001.zip › cells-1621181-supplementary.pdf]

Patients for analysis	OvCA PBL N_{total}=17	OvCA MAL N_{total}=18	OvCA TIL N_{total}=9	HD PBL N_{total}=14
Age<br>Median (range)	60 (32-86)	61 (32-86)	57 (32-66)	59 (26-64)
Sex	All female	All female	All female	All female

Table S1: Patient characteristics of patients included in the study.
Age and sex of the donors included in the study are depicted. The cohort included peripheral blood (PBL), malignant ascites (MAL) and tumor-infiltrating lymphocytes (TIL) of patients with ovarian cancer (OvCA) as well as PBL from healthy volunteers (HD).

Patient	Diagnosis	FIGO stage	Lymph node metastasis	Meta-stasis	pT stage	Residual tumor after surgery	Primary/ Recurrent	Material
Pat#1	HGSOC	IIIC	N1	M0	pT2a	> 1cm	primary	PBL, MAL
Pat#2	HGSOC	IIIC	N1	M0	pT3c	microscopic	recurrent	PBL, MAL
Pat#3	HGSOC	IVb	N1	M1	pT3c	> 1cm	primary	PBL, MAL
Pat#4	HGSOC	III	N1	M0	pT3c	< 1cm	primary	PBL*, MAL
Pat#5	HGSOC	IIIC	Nx	M0	pT3b	< 1cm	primary	PBL, MAL
Pat#6	HGSOC	IIIC	N1	M0	pT3c	< 1cm	primary	PBL, MAL
Pat#7	HGSOC	IIIC	N0	M0	pT3c	microscopic	primary	PBL, MAL
Pat#8	HGSOC	IV	N1	M1	pT3c	<1 cm	primary	PBL, MAL
Pat#9	HGSOC	IIIC	N0	M0	pT3c	microscopic	primary	PBL, MAL, TIL
Pat#10	HGSOC	IIIC	N1	M0	pT3c	microscopic	primary	PBL, MAL, TIL
Pat#11	HGSOC	IIIC	N1	M0	pT3c	microscopic	recurrent	PBL, MAL, TIL
Pat#12	HGSOC	IIIC	N1	M0	pT3c	<1 cm	primary	PBL*, MAL*, TIL
Pat#13	HGSOC	IIIC	Nx	M0	pT3c	> 1cm	primary	PBL*, MAL*, TIL
Pat#14	HGSOC	IIIC	N1	M0	pT3c	microscopic	primary	PBL, MAL, TIL
Pat#15	HGSOC	IV	N0	M1	pT3c	< 1cm	primary	PBL, MAL, TIL
Pat#16	HGSOC	IIIC	N0	M0	pT3c	1 cm	primary	PBL, MAL, TIL
Pat#17	HGSOC	IIIC	N0	M0	pT3c	> 1cm	primary	PBL, MAL, TIL
Pat#18	HGSOC	IIIC	N1	M0	pT3c	microscopic	primary	PBL, MAL, TIL

* Material has been excluded for checkpoint analyses from the study .

Table S2: Clinical characteristics of patients included in the study.
Clinical data such as diagnosis (HGSOC = high grade serous ovarian cancer), FIGO stage, TNM characteristics, tumor residual after surgery, tumor status (primary/recurrent) and material aquired are depicted in this table.

Antibody	Clone
anti-CD3	OKT3
anti-CD4	RPA T4
anti-CD8	RPA T8
anti-EPCAM	9C4
anti-γδ TCR	B1
anti-Vδ1 TCR	REA173
anti-Vδ2 TCR	REA771
anti-CD45RA	HI100
anti-CD27	O323
anti-CD19	HIB19
anti-CD56	HCD56
anti-PD-1	EH12.2H7
anti-TIGIT	A15153G
anti-TIM-3	F38-2E2
anti-CD39	A1
anti-CD73	AD2
anti-Ox40	Ber-ACT35

Table S3: Antibodies and clones used in the study.
Applied antibodies with their respective clones are depicted in this table.

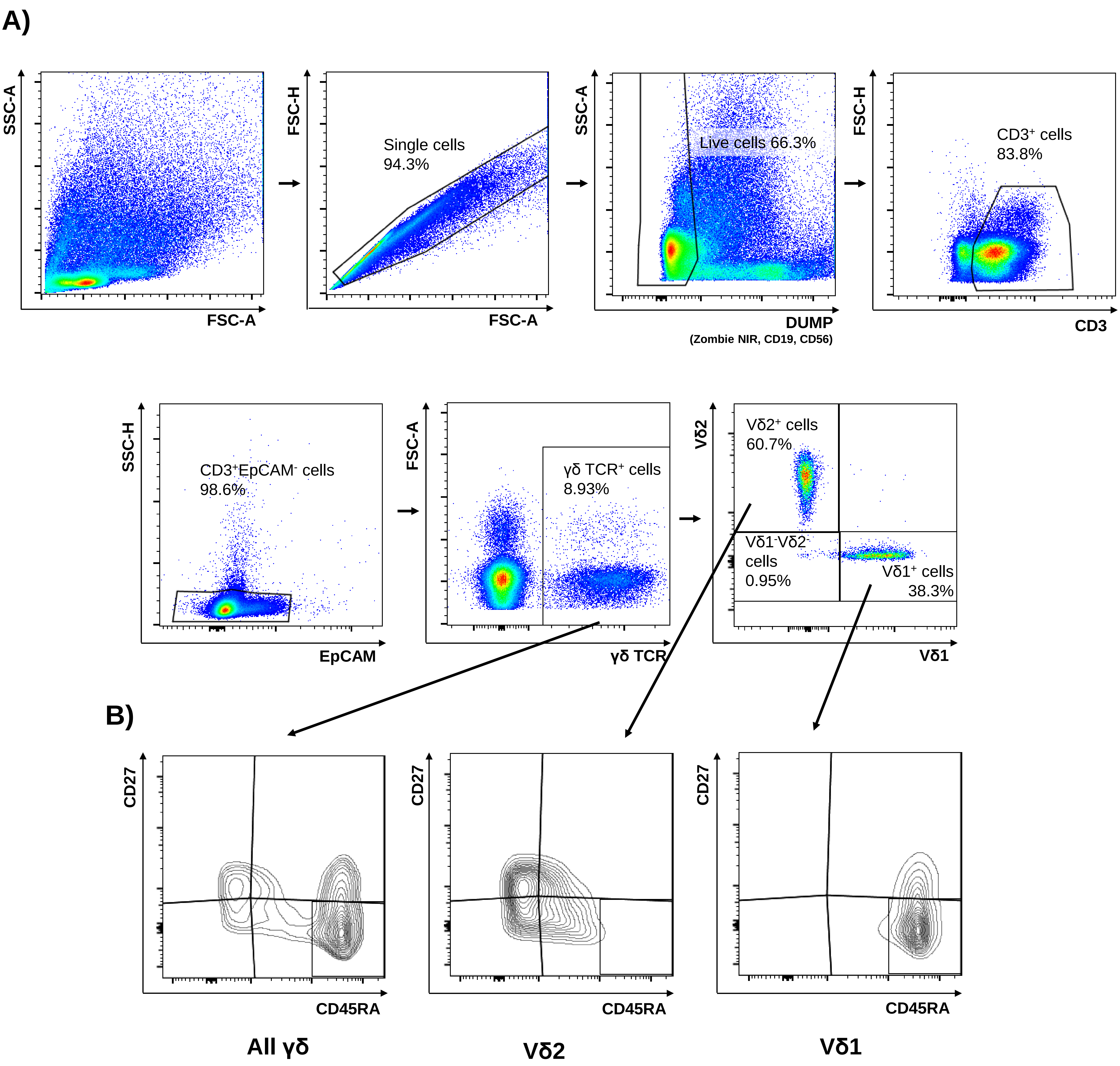

Figure S1: Flow cytometry gating strategy to identify (Vδ1 and Vδ2) γδ T cells. Peripheral blood- (PB) and malignant ascites- (MA) derived mononuclear cells and tumor infiltrating lymphocytes (TIL) were analyzed via the same gating strategy. For each sample, one FACS tube was stained according to the gating strategy listed below, as well as fluorescence minus one (FMO) controls for the single molecules. **(A)** After exclusion of doublets and cell debris, the living cells of interest were isolated via DUMP-negative gating. The DUMP channel included Zombie NIR, CD19 and CD56 in all tubes. The DUMP-gating excluded B-lymphocytes and NK cells together with the dead cells. Next, $CD3^+$ T cells were gated on, followed by an exclusion of $EpCAM^+$ ovarian cancer cells. After isolating the γδ T cells, this population was further divided into the Vδ1 and Vδ2 γδ T cell subpopulation regarding the expression of the corresponding receptors. **(B)** Gating of the differentiation markers CD27 and CD45RA on all γδ T cells (left), Vδ1 (middle) and Vδ2 cells (right).

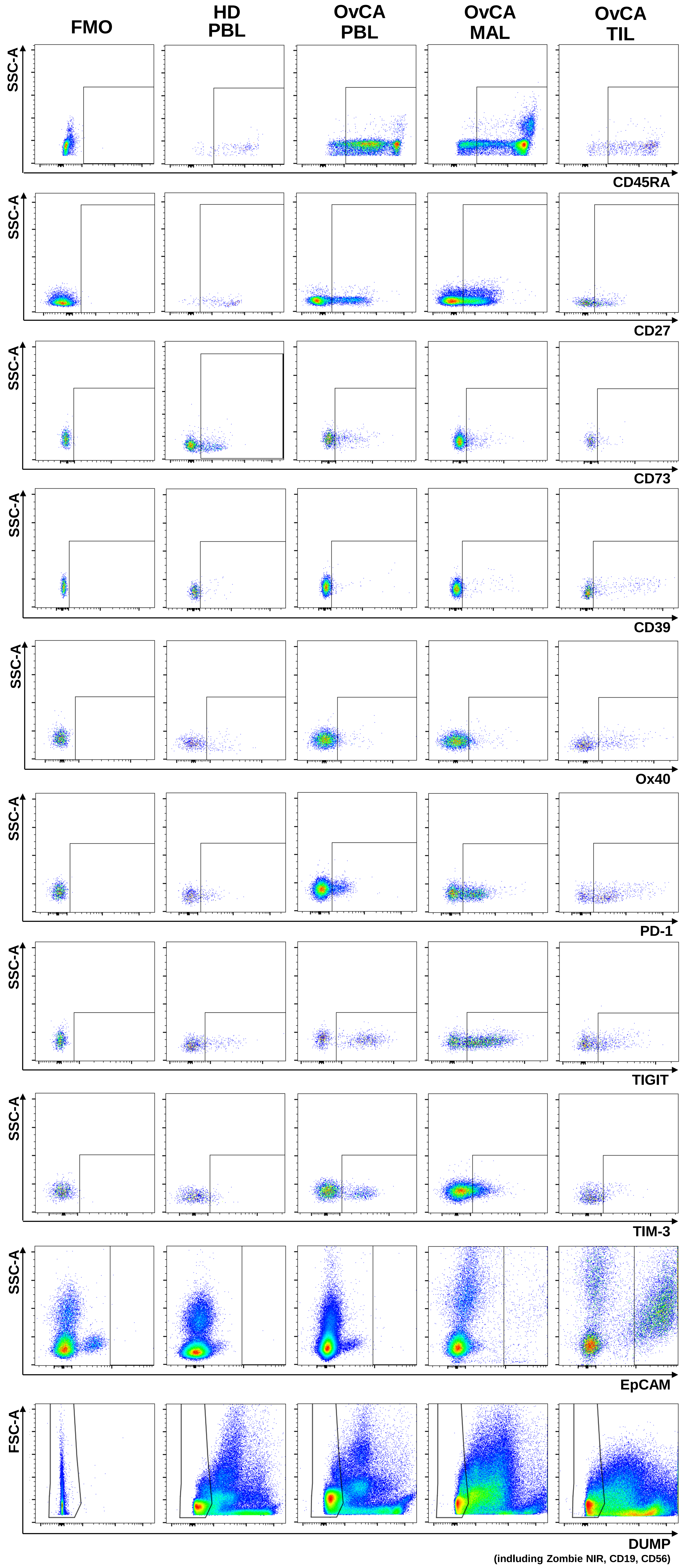

Figure S2: Fluorescence minus one (FMO) flow cytometry data to identify positive expression. DUMP-gating, differentiation and expression of ectonucleotidases, activation markers and co-inhibitory receptors (second to left panel: peripheral blood (PB) from healthy donors (HD), middle panel: PB from ovarian cancer (OvCA) patients; second to right panel: malignant ascites (MA) from OvCA patients, right panel: tumor-infiltrating lymphocytes (TIL) from OvCA patients) were gated with contemplation of FMOs (left panel), identifying the cutoff of positive expression.

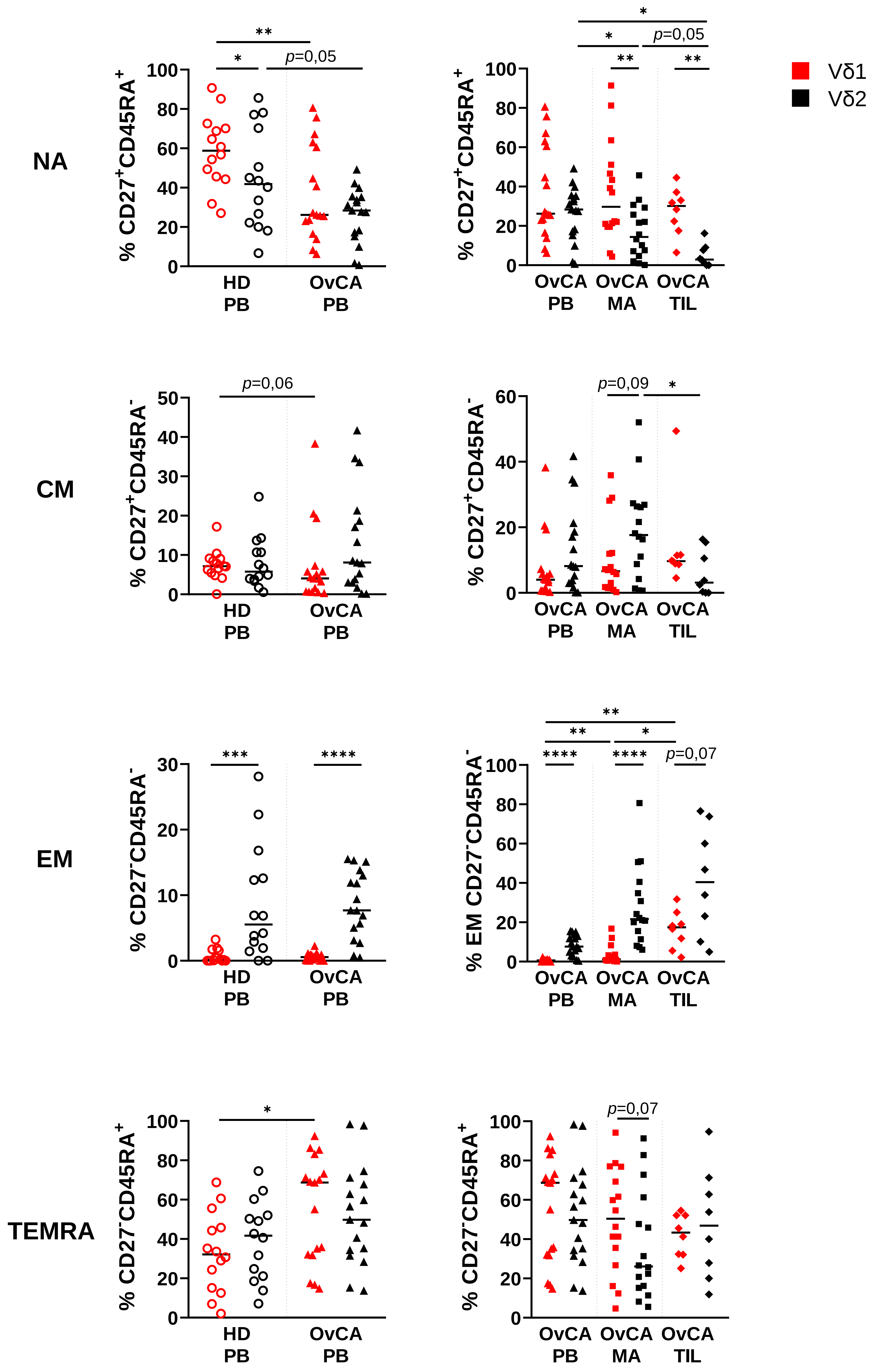

Figure S3: Differentiation status of γδ T cells.
The differentiation of Vδ1 (red) and Vδ2 (black) T cells into the naive (NA, $CD27^{+}CD45RA^{+}$) central memory (CM, $CD27^{+}CD45RA^{-}$), effector memory (EM, $CD27^{-}CD45RA^{-}$) and terminally differentiated effector memory (TEMRA, $CD27^{-}CD45RA^{+}$) cells was compared between the peripheral blood (PB) of healthy donors (HD; circles, n=14) and the PB of ovarian cancer (OvCA) patients (triangles, n=17), as well as between PB, malignant ascites (MA, squares, n=16) and tumor-infiltrating lymphocytes (TIL, diamonds, n=8) of OvCA patients. *P* values were obtained by the Mann-Whitney-Test and Wilcoxon matched-pairs signed-rank test. *P<0.05, **P<0.01, ***P<0.001, ****P<0.0001.

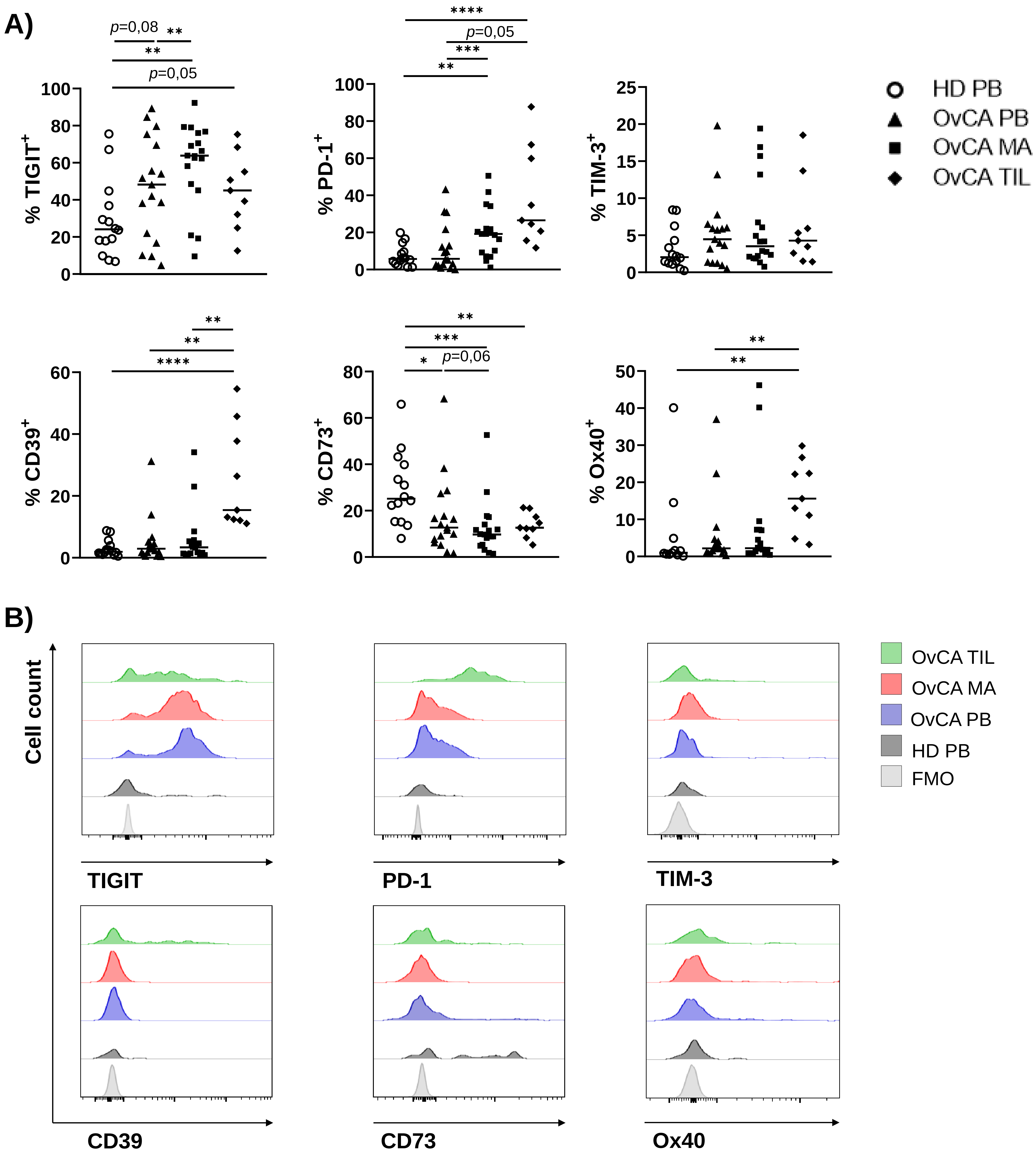

Figure S4: Expression of co-inhibitory receptors, ectonucleotidases and activation markers on γδ T cells.
The expression of TIGIT, PD-1, TIM-3, CD39, CD73 and Ox40 was compared on γδ T cells. **(A)** Summary data show the frequency of positive cells within the peripheral blood (PB) from healthy donors (HD, white circles, n=14), and PB (triangles, n=17), malignant ascites (MA, squares, n=18), and tumor-infiltrating lymphocytes (TILs, diamonds, n=9) of ovarian cancer (OvCA) patients. *P* values were obtained by the Wilcoxon matched-pairs signed-rank test and by the Mann-Whitney-Test. **P*<0.05, ***P*<0.01, ****P*<0.001, *****P*<0.0001. **(B)** Histograms show the median fluorescence intensity of the analyzed molecules in the respective compartments vs. FMO control.

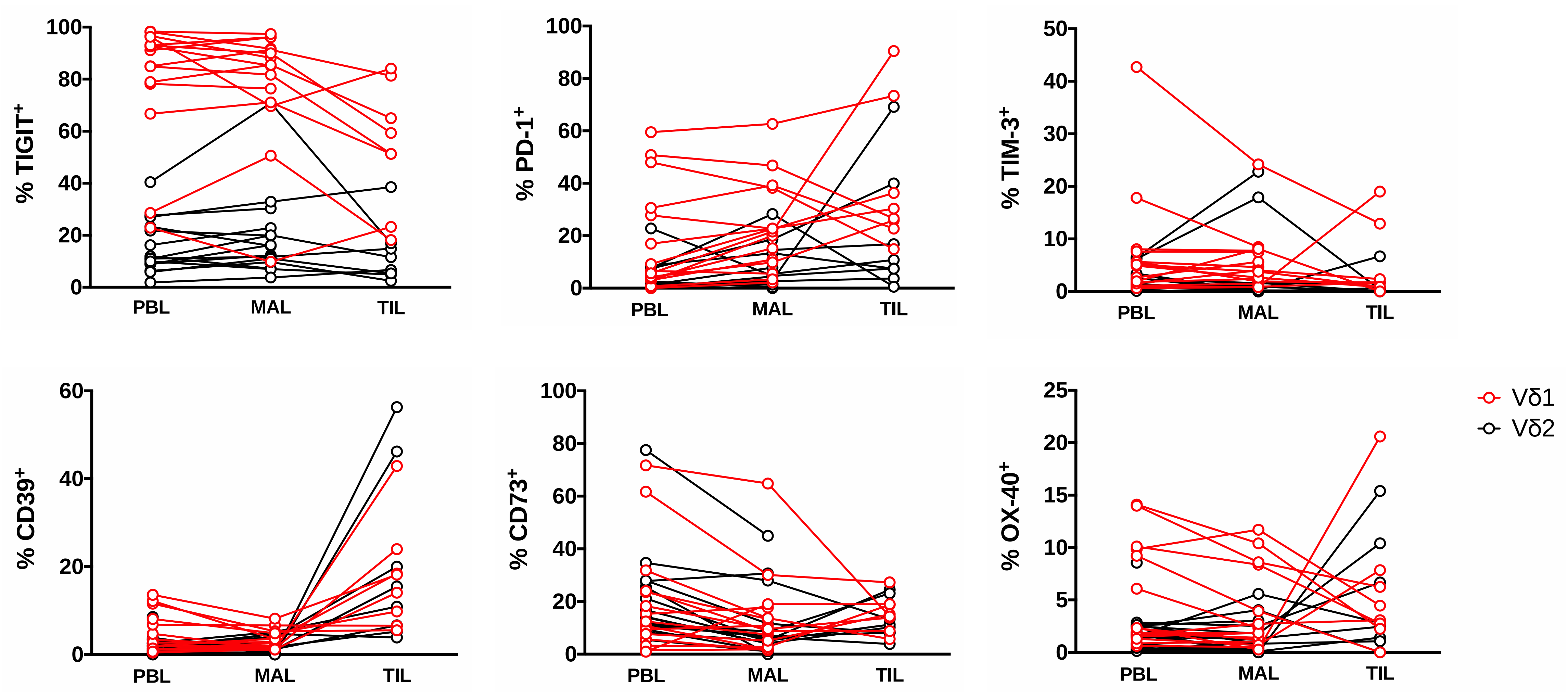

Figure S5: Expression of co-inhibitory receptors, ectonucleotidases and activation markers on Vδ1 and Vδ2 T cells.

The expression of PD-1, TIM-3, CD39, CD73 and Ox40 was compared on Vδ1 (red) and Vδ2 (black) T cells within 8 triple matched samples: peripheral blood (PB, triangles), malignant ascites (MA, squares), and tumor infiltrating-lmphocytes (TIL, diamonds) and within 7 double matched specimens (PB and MA) of ovarian cancer (OvCA) patients. The connecting lines indicate the expression in the different tissues from one patient. *P* values were obtained by the Wilcoxon matched-pairs signed-rank test. **P*<0.05, ***P*<0.01, ****P*<0.001, *****P*<0.0001.

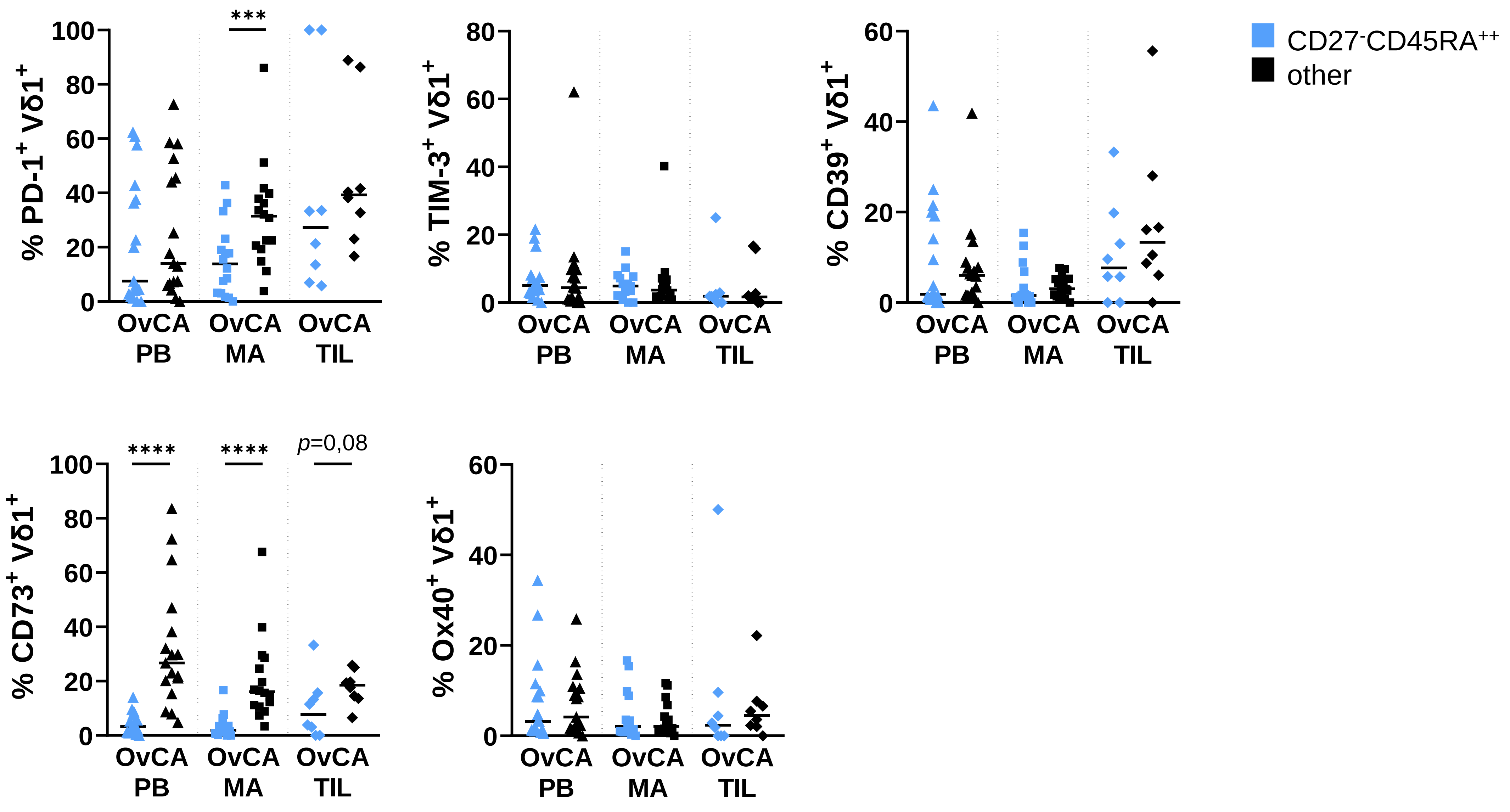

Figure S6: Expression of co-inhibitory receptors, ectonucleotidases and activation markers on Vδ1 CD27^{-}CD45RA^{high} T cells.

The expression of PD-1, TIM-3, CD39, CD73 and Ox40 was compared between subsets of Vδ1 T cells in peripheral blood (PB, triangles, n=17), malignant ascites (MA, squares, n=16), and tumor-infiltrating lymphocytes (TILs, diamonds, n=8) of ovarian cancer (OvCA) patients. Summary data show the expression of the markers on CD27^{-}CD45RA^{high} cells (light blue) vs. all other differentiation stages (black). *P* values were obtained by the Wilcoxon matched-pairs signed-rank test. **P*<0.05, ***P*<0.01, ****P*<0.001, *****P*<0.0001.

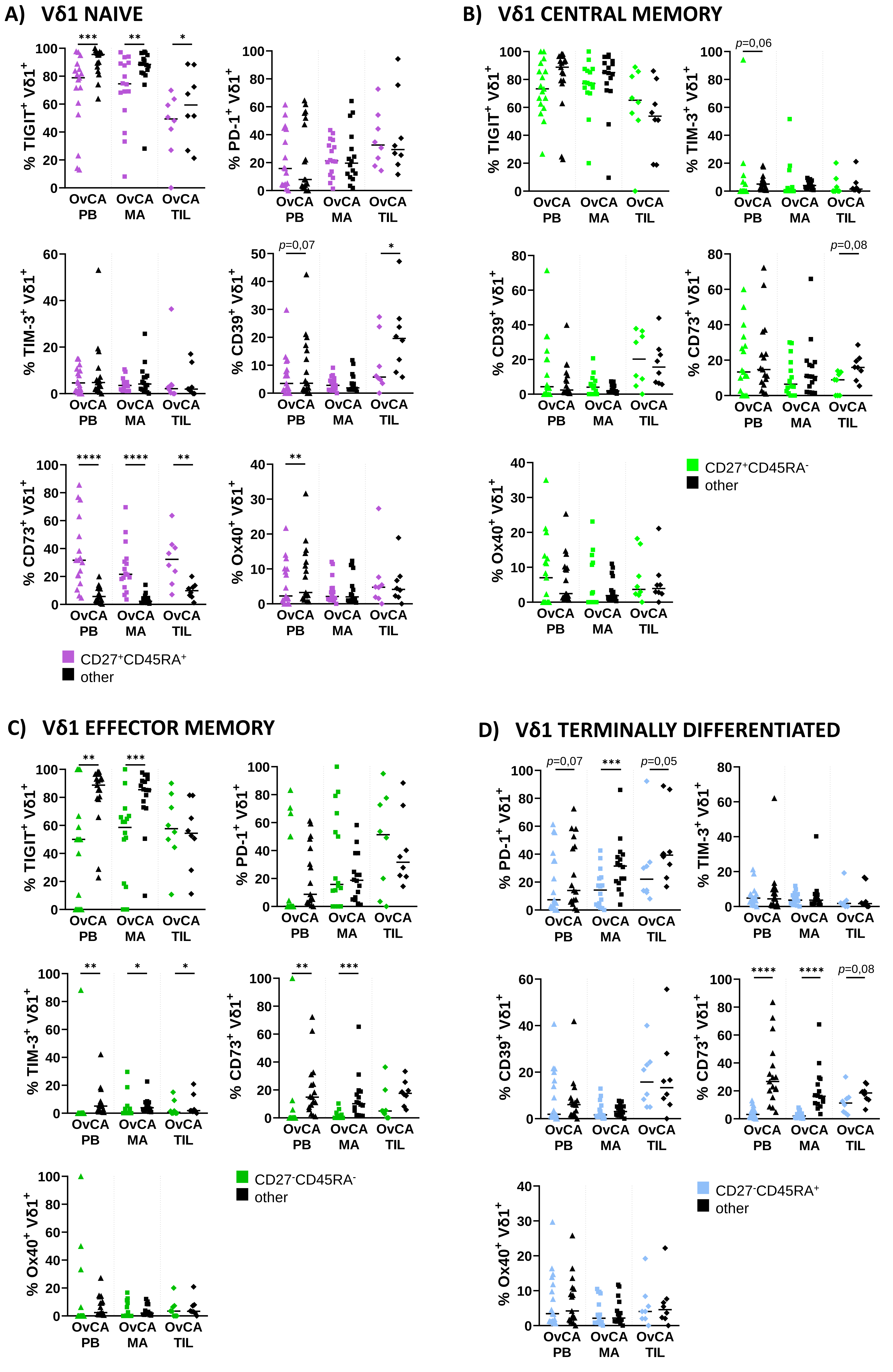

Figure S7: Expression of co-inhibitory receptors, ectonucleotidases and activation markers on Vδ1 differentiation stages.
The expression of TIGIT, PD-1, TIM-3, CD39, CD73 and Ox40 was compared between subsets of Vδ1 T cells in peripheral blood (PB, triangles, n=17), malignant ascites (MA, squares, n=16), and tumor-infiltrating lymphocytes (TILs, diamonds, n=8) of ovarian cancer (OvCA) patients. Summary data show the expression of the markers on **(A)** naïve (purple), **(B)** central memory (light green), **(C)** effector memory (dark green), and **(D)** terminally differentiated (blue) cells vs. all other respective differentiation stages (black). *P* values were obtained by the Wilcoxon matched-pairs signed-rank test. **P*<0.05, ***P*<0.01, ****P*<0.001, *****P*<0.0001.

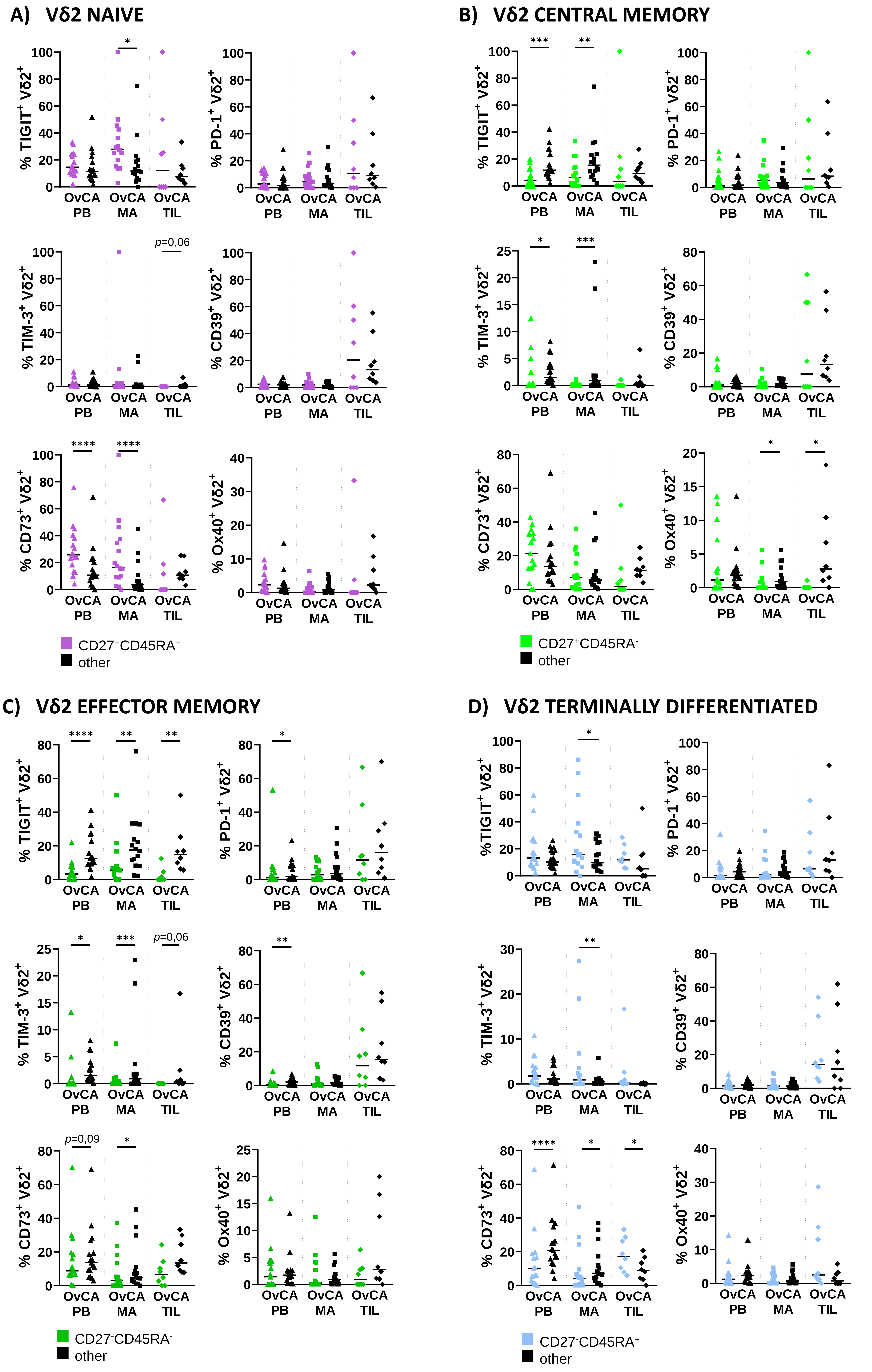

Figure S8: Expression of co-inhibitory receptors, ectonucleotidases and activation markers on Vδ2 differentiation stages.
The expression of TIGIT, PD-1, TIM-3, CD39, CD73 and Ox40 was compared between subsets of Vδ2 T cells in peripheral blood (PB, triangles, n=17), malignant ascites (MA, squares, n=16), and tumor-infiltrating lymphocytes (TILs, diamonds, n=8) of ovarian cancer (OvCA) patients. Summary data show the expression of the markers on **(A)** naïve (purple), **(B)** central memory (light green), **(C)** effector memory (dark green), and **(D)** terminally differentiated (blue) cells vs. all other respective differentiation stages (black). *P* values were obtained by the Wilcoxon matched-pairs signed-rank test. **P*<0.05, ***P*<0.01, ****P*<0.001, *****P*<0.0001.

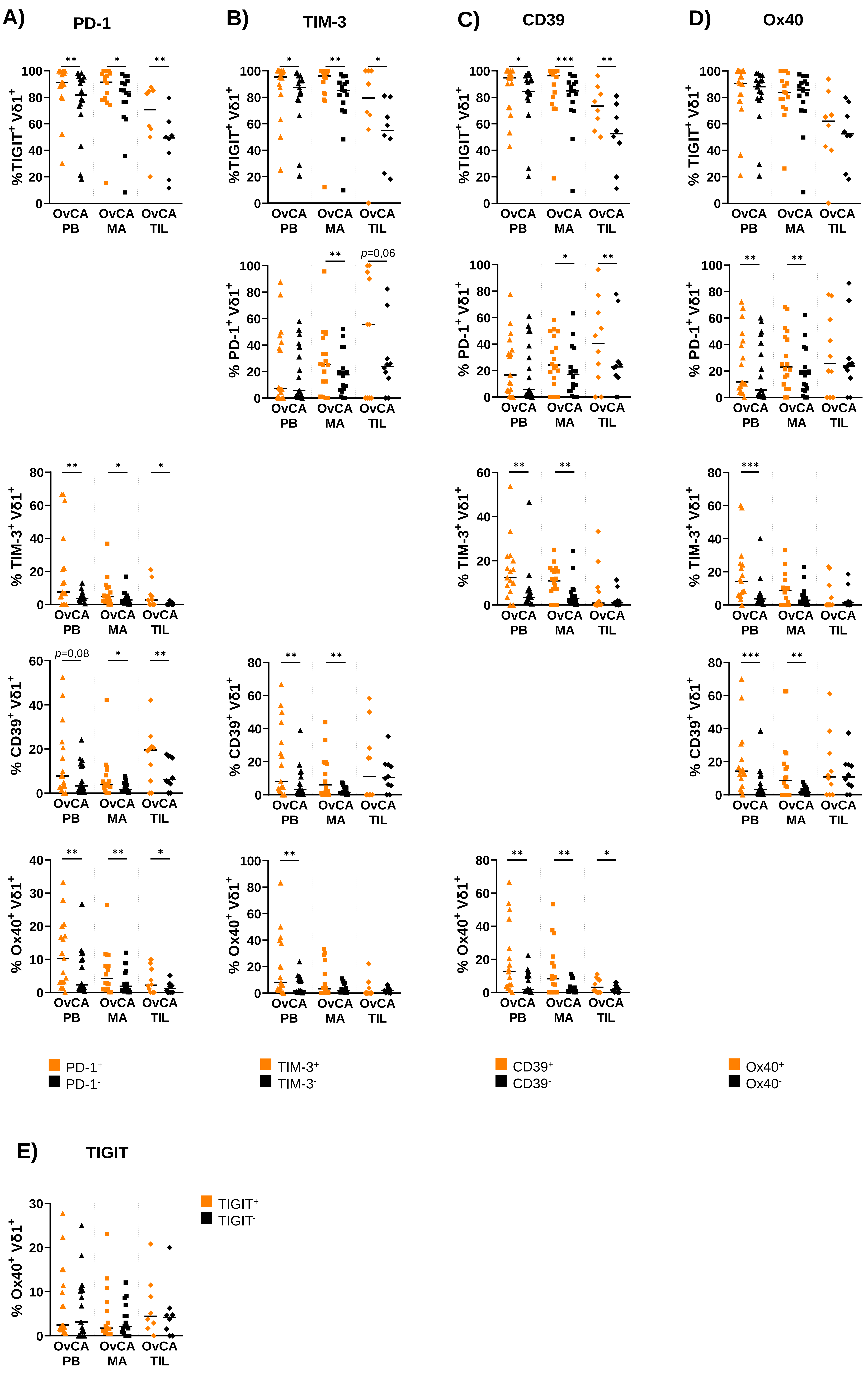

Figure S9: Co-expression of co-inhibitory receptors, ectonucleotidases and activation markers on Vδ1 T cells.

The co-expression of TIGIT, PD-1, TIM-3, CD39, CD73 and Ox40 was compared on Vδ1 T cells in peripheral blood (PB, triangles, n=17), malignant ascites (MA, squares, n=16), and tumor-infiltrating lymphocytes (TILs, diamonds, n=8) of ovarian cancer (OvCA) patients. Summary data show the expression of the respective markers on **(A)** PD-1$^+$, **(B)** TIM-3$^+$, **(C)** CD-39$^+$, and **(D)** Ox40$^+$ **(E)** TIGIT$^+$ cells (orange) vs. the cells not expressing the underlying molecules (black). *P* values were obtained by the Wilcoxon matched-pairs signed-rank test. **P*<0.05, ***P*<0.01, ****P*<0.001, *****P*<0.0001.
